# MXenes as Emerging Materials: Synthesis, Properties, and Applications

**DOI:** 10.3390/molecules27154909

**Published:** 2022-08-01

**Authors:** Ubaid Ur Rahman, Muhammad Humayun, Usman Ghani, Muhammad Usman, Habib Ullah, Adil Khan, Nashwa M. El-Metwaly, Abbas Khan

**Affiliations:** 1 Department of Chemistry, Abdul Wali Khan University, Mardan 23200, Pakistan; ubaidur733@gmail.com (U.U.R.); usman511ug@gmail.com (U.G.); adilmkd77@gmail.com (A.K.); 2 Wuhan National Laboratory for Optoelectronics, School of Optical & Electronics Information, Huazhong University of Science and Technology, Wuhan 430074, China; 2017511018@hust.edu.cn; 3 Interdisciplinary Research Center for Hydrogen and Energy Storage (IRC-HES), King Fahd University of Petroleum & Minerals (KFUPM), Dhahran 31261, Saudi Arabia; muhammadu@kfupm.edu.sa; 4 Department of Materials Science & Engineering, Chungnam National University, 99 Daehak-ro, Yuseong-gu, Daejeon 34134, Korea; habib.ullah@uskt.edu.pk; 5 Department of Chemistry, University of Sialkot, Sialkot 51040, Pakistan; 6 Department of Chemistry, Faculty of Applied Science, Umm-Al-Qura University, Makkah 21955, Saudi Arabia

**Keywords:** MXenes, max phases, MXenes composites, 2D materials, layered materials

## Abstract

Due to their unique layered microstructure, the presence of various functional groups at the surface, earth abundance, and attractive electrical, optical, and thermal properties, MXenes are considered promising candidates for the solution of energy- and environmental-related problems. It is seen that the energy conversion and storage capacity of MXenes can be enhanced by changing the material dimensions, chemical composition, structure, and surface chemistry. Hence, it is also essential to understand how one can easily improve the structure–property relationship from an applied point of view. In the current review, we reviewed the fabrication, properties, and potential applications of MXenes. In addition, various properties of MXenes such as structural, optical, electrical, thermal, chemical, and mechanical have been discussed. Furthermore, the potential applications of MXenes in the areas of photocatalysis, electrocatalysis, nitrogen fixation, gas sensing, cancer therapy, and supercapacitors have also been outlooked. Based on the reported works, it could easily be observed that the properties and applications of MXenes can be further enhanced by applying various modification and functionalization approaches. This review also emphasizes the recent developments and future perspectives of MXenes-based composite materials, which will greatly help scientists working in the fields of academia and material science.

## 1. Introduction

Global attention is currently focused on energy and environmental challenges. Electric vehicles and stationary batteries both require high-capacity energy conversion/storage systems, while the present energy conversion and storage techniques such as batteries, water splitting, and supercapacitors are being advanced. Thus, further research is required because simultaneous high energy conversion and power density is difficult to attain, and rechargeable technologies are expensive and limited in application [1,2,3,4,5,6]. Because of their unique physicochemical features, two-dimensional (2D) materials such as MXenes have received a lot of interest. MXenes are also very interesting in this context as they are relatively safe, have broad interlayer spacing, are environmentally flexible, and have excellent biocompatibility [7]. MXenes, a broad family of the 2D transition-metal carbides or nitrides, was recently developed by Drexel University researchers. The generic formula for MXenes is M_n+1_X_n_T*_x_* (n = 1–3), where M stands for the transition metals (i.e., Ti, Zr, Ta, Nb, V, Mo, etc.) and X represents the carbon or nitrogen elements. Typically, MXenes are synthesized from the layered-structure MAX-phase bulk ceramic using fluoride-based chemicals to selectively etch (usually groups IIIA and IVA) layers, and their basal faces are frequently terminated with special surface functionalities (T*_x_*), such as O, OH, and F. Due to their exceptional conductivity and the presence of abundant surface functionalities, MXenes with highly adjustable structural and chemical forms can perform both as fundamental active materials and carriers of additional functional materials for a variety of applications, such as photodetectors [8], flame-retardant polymer materials [9], water purification [10,11], energy conversion and storage [12,13,14], sensors, electro-magnetic interference shielding, gas separation, biomedical imaging and therapy, and catalysis [15,16,17,18].

As a result of their wide range of potential in the realms of energy conversion and storage, MXenes have astonished the scientific community [19]. In comparison to other 2D materials [20,21], MXenes exhibit good hydrophilicity, film-forming performance, and electrical conductivity. They are commonly used in supercapacitors, electro-magnetic radiation shielding, and lithium-ion batteries [22].

Due to their flexible surface chemistry, metallic conductivity, redox capability, and graphene-like structure, MXenes are possible 2D materials for a wide range of applications. MXenes are usually comprised of nontoxic elements such as Ti, C, and N, and could be used for environmental applications [23]. Titanium carbide (Ti_3_C_2_) was the first MXene reported at Drexel University. The Ti_2_C, Nb_2_C, V_2_C, Ti_3_CN, Mo_2_C, and Ta_4_C_3_ members have been prepared successfully among the several theoretically predicted Mxenes [24]. Ti_3_C_2_T*_x_* is one of the most frequent and investigated MXenes among them [25]. Since then, researchers have been studying MXenes in order to learn more about their properties and applications. MXenes are an attractive possibility for replacing current materials in energy storage, biological, and environmental purposes such as drinking water and electro-magnetic shielding. MXenes also have wide range of uses as catalysts for oxygen reduction reactions (ORR) [26], oxygen evolution reactions (OER) [27], CO oxidation, hydrogen storage material for dehydrogenation, and hydrogen evolution reactions. They are also used in sensor technologies, such as electrochemical biosensors, gas sensors, and macromolecule and cell detection [28]. In addition, MXenes with a large negative zeta potential are mixable in a wide range of solvents, including C-Ns and polymeric materials, allowing for the creation of virtually limitless composites with a wide range of characteristics [29].

**Figure 1:**
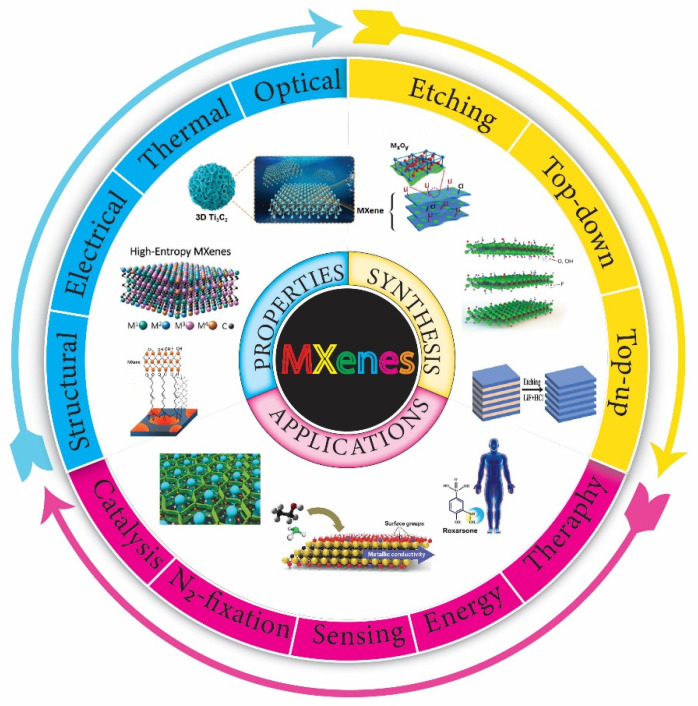
Scheme for the synthesis, properties, and applications of MXenes.

However, the discovery of an alternative LiF salt precursor in 2014 ushered in a new era in MXenes research, with a slew of new uses emerging since then. MXenes could be used in rechargeable battery electrodes, supercapacitors, electro-magnetic interference shielding, sensors, catalysis, photocatalysis, transparent conducting films, flexible composites with high strength, crude oil, heavy-metal adsorption agents, and many other applications [30]. For example, electrochemical energy conversion and storage is critical for developing renewable and clean energy sources to address the energy shortage issues. As a result, there is a strong need to keep up with recent MXene electrochemistry and energy application advancements. The virtue of MXenes include their discovery, synthesis, family types, and characteristics. Following that, in the current review, we reviewed the fabrication, properties, and potential applications of MXenes. In addition, the structural, optical, electrical, thermal, chemical, and mechanical properties of MXenes have been discussed in detail. Furthermore, the potential applications of MXenes in the area of photocatalysis, electrocatalysis, nitrogen fixation, gas sensing, cancer therapy, and supercapacitors have also been outlooked (Figure 1). Finally, the conclusions and future perspectives for MXene-based materials are explored.

## 2. Synthesis Methods

**Figure 2:**
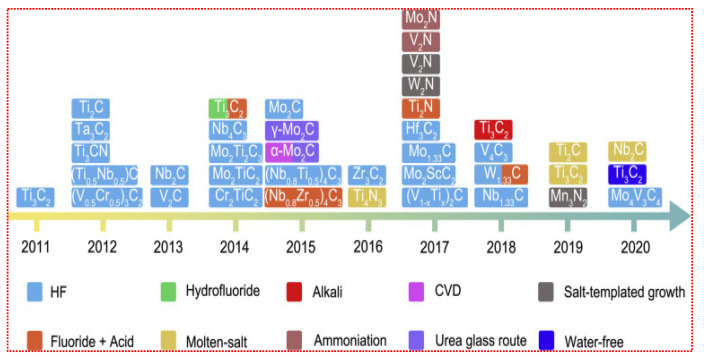
MXenes synthesis routes timeline in last decade. Reproduced with permission from ref. [31] Copyright 2021, Elsevier.

The development of MXene synthesis pathways had a significant impact on their electrical characteristics, physicochemical features, and a wide range of applications. In general, there are three types of MXene synthesis techniques: Etching, top-down, and bottom-up. In the etching method, the “A” elements from the MAX phases of the parent three-dimensional (3D) layer result in the MXenes layered structures. Until now, the most prevalent approach for synthesizing MXenes has been recognized as the top-down strategy (see Figure 2). Unlike the top-down fabrication approach, which usually requires a large number of precursor materials, the bottom-up synthesis fabrication route requires few organic or inorganic molecules/atoms when carefully constructing the structure. Using a crystal growth method, the precursors can be assembled into a definite 2D order constituting the MXene structures. The advantages of the bottom-up approach allow for exquisite control of the size distribution, shape, and surface terminal functionalities of MXenes in an appropriate way [31].

Normally, MXenes refers to M_n_X_n–1_ (n = 2, 3, and 4) layers formed by removing interlayer “A” atoms from the metal-ceramic MAX phase (where M stands for the transition metals, A is the group IIIA or IVA elements, and X represents the C and N elements). The M and X atoms stack to form a hexagonal lattice in the MAX phase, with the atoms of X occupying the M octahedral cage center shared by its edges. When the atoms of A are removed from the layer of M_n_X_n–1_, the hexagonal-lattice of MX, rather than the cubic lattice, is preserved. Hence, the layer of M_n_X_n–1_ can be produced by removing the A-atoms. As with its predecessor MAX, MXenes thin sheets are frequently oriented horizontally. The bulk of MXenes have good mechanical features and are likely to be quite durable [32].

### 2.1. Etching Method

MXenes can be prepared in a number of different ways. Due to changes in their etching methods, various terminal functionalities can be attached to the M atoms to complete their coordination spheres, so as to lower their surface Gibbs free energy. As a result, the MXenes surface features have a great influence on their fabrication. Herein, different types of the preparation methods are discussed.

#### 2.1.1. Etching with Hydrofluoric Acid (HF)

**Figure 3:**
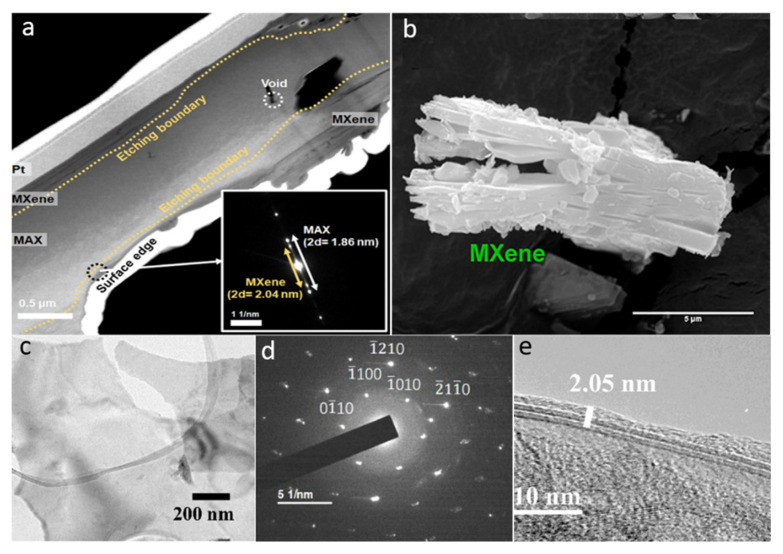
(**a**) STEM micrograph of the bulk-etched MAX phase Ti_3_AlC_2_ for 3 h and the corresponding SAED pattern as inset. Reproduced with permission from ref. [36] Copyright 2021, The American Chemical Society. (**b**) The SEM micrograph of CoF_3_/MAX without any AlF_3_·3H_2_O impurity. Reproduced with permission from ref. [38] Copyright 2019, The American Chemical Society. (**c**) TEM micrograph, (**d**) the corresponding SAED pattern, and (**e**) HR-TEM micrograph of the MS-Ti_3_C_2_T*_x_*. Reproduced with permission from ref. [42] Copyright 2022, The American Chemical Society.

Due to the in-depth research on MXenes, etching procedures have been widely employed, especially the HF acid etching method, which is still the most commonly used procedure. Naguib et al. [33] proposed the fabrication of the Ti_3_AlC_2_ MAX phase via HF acid etching in 2011. Using a simple displacement process, the HF acid removes Al layers from the Ti_3_AlC_2_ MAX phase with H_2_ production. In addition, the reaction of deionized water with the HF acid solution produces Ti_3_C_2_T*_x_* (where T represents the -O, -F, and -OH) as well as H_2_. The HF acid etching was used to successfully strip the Ti_2_AlC, (Ti_0.5_Nb_0.5_) 2AlC, Ti_3_AlCN, Ta_4_AlC_3_, (V_0.5_Cr_0.5_) 3AlC_2_, Nb_2_AlC, and a series of the MAX complexes of Zr_3_Al_3_C_5_, Ti_3_SiC_2_, and Mo_2_Ga_2_C, into Mxenes [34]. The HF acid etching has continuously been the most useful synthesis process for MXenes materials from 2011 to date. In the HF acid etching process, the time period, temperature, and F ions density play an important role in the design of high-quality MXenes layers. Alhabeb et al. [35] confirmed that Ti_3_C_2_T_X_, with high concentration HF acid, produces an excellent layered structure, which is difficult to achieve with other acid solutions. MXenes obtained via the HF acid etching approach retained their unique surface characteristics, along with the –O, –OH, and –F functionalities. Recently, Kim et al. [36] studied the etching behavior of MAX-phase Ti_3_AlC_2_ in various etching agents at the atomic scale employing ion-beam and electron microscopy. They investigated the change in the structure of the Ti_3_AlC_2_ phase as a function of the etching agents and etching time, revealing that the edge Al atoms at the mid layers of MAX-phase Ti_3_AlC_2_ are not etched, despite the interaction with the HF etchant. Further, etching of the grain boundary occurred in the HF etchant. As revealed in the STEM micrograph (Figure 3a), employing the bulk etching technique, the etching of Ti_3_AlC_2_ for 3 h displayed numerous etched regions. Further, the SAED pattern (inset Figure 3a) reveals the expansion of d-spacing of the MAX-phase Ti_3_AlC_2_ (i.e., 0.97 nm) to 1.02 nm, confirming the successful conversion to Ti_3_C_2_T*_x_* MXene at the etching boundary.

#### 2.1.2. Modified Acid Etching Approach

Acid fluoride solutions have corrosive and poisonous properties, and due to these reasons, researchers are finding techniques to prevent HF acid from being used directly to extract the Al layers from MAX phases. The foremost approach, known as original-location HF acid etching, replaces the HF acid with fluoride salts (i.e., LiF, NH_4_HF_2_, FeF_3_, KF, and NaF) and HCl [37]. Usually, during the synthesis of MXenes via the etching of Al or Ga layers of the MAX phase by HF acid, an undesired byproduct (i.e., AlF_3_·3H_2_O) is formed. To synthesize MXenes free of this impurity, it is necessary to illuminate the factors that initiate its formation. Thus, modified etching approaches are widely employed. For instance, Cockreham et al. [38] deduced the conditions that led to the formation of the byproduct AlF_3_·3H_2_O, while proceeding the etching process with cobalt fluorides (i.e., CoF_2_/CoF_3_). The SEM micrograph of the CoF_3_/MAX sample (Figure 3b) do not show any AlF_3_·3H_2_O impurity. The MXenes interlayer distance formed via the modified acid-etching approach is improved because of the cation’s intercalation, which minimizes the inner force between layers, and potentially delaminates the material layers during ultrasonication. As a result, this approach shortens the previously noted time-consuming multi-step synthesis process, enabling the synthesis of few-layer MXene in a single step [39].

#### 2.1.3. Modified Fluoride-Based Acid Etching

Researchers have worked hard to find better means to remove the atom layers from MAX in order to prevent the significant toxicity that HF etching causes. Apart from HF, etching MAX precursors can also be performed with a mix of fluoride salts (i.e., KF, NaF, LiF, and NH_4_F) and strong acids. Fluoride salts and strong acids have been discovered to selectively etch atoms and led to the cation’s intercalation (i.e., K^+^, Na^+^, Li^+^, and NH_4_^+^) in situ. The introduction of water between MXene layers increases the interlayer space while lowering MXene layer contact. It is worth noting that the amount of fluoride salt and strong acid in the final MXene fragments can affect their quality and size. For example, the multilayered Ti_3_C_2_ generated by the clay approach (5M LiF/6M HCl) requires a subsequent sonication step for delamination into single flakes, which frequently results in tiny defective MXene flakes [40].

#### 2.1.4. Molten Salts Etching

MXene can also be produced by heating the MAX phases, such as Ti_4_AlN_3_, in a molten fluoride salt mixture (i.e., LiF, NaF, KF (29:12:59 weight ratio %)) at 550 °C under argon shielding to make Ti_4_N_3_. The etching procedure can be completed in 30 min. Because the Ti_n_N_n–1_ has a lower stability than Ti_n_C_n–1_, it can be dissolved in HF or other fluoride-based acids used as the etching agent. As a result, the molten-salts etching method has the benefit of a relatively quick processing time. Additional cleaning (washing with DI H_2_O and H_2_SO_4_) and delamination (in TBAOH solution) are compulsory. The crystallinity of delaminated Ti_4_N_3_ is lower than that of the MXene obtained via the HF etching, as indicated by the XRD patterns of the resultant Ti_4_N_3_. The TiO_2_ phase is also visible in the obtained product. In comparison to the HF and fluoride-based acids etching, the molten salts etching process has the benefit of producing MXenes with limited stability in the HF or fluoride-based acids solution. This technique, on the other hand, has the following disadvantages: (a) The etching process consumes a significant amount of heat and energy; (b) the resultant MXenes reveal low purity and crystallinity; (c) the resultant MXenes have abundant surface defects and vacancies [41]. Very recently, Liu et al. [42] reported the synthesis of MS-Ti_3_C_2_T*_x_* via the intercalation of the tetrabutylammonium hydroxide (TBAOH), followed by layer separation via sonication. The as-obtained Cl-terminated MS-Ti_3_C_2_T*_x_* was utilized as an anode in a Li-ion battery, which achieved a high specific capacity and exceptional rate capability. The TEM micrograph (Figure 3c) reveals clear Ti_3_C_2_T*_x_* nanosheets with distinct edges, with a lateral size of ~600 nm.

The SAED pattern (Figure 3d) reveals sharp reflections corresponding to the hexagonal crystal symmetry, suggesting that the TBAOH treatment did not altered the crystallinity of Ti_3_C_2_T*_x_*. The HR-TEM micrograph (Figure 3e) confirmed a 2.05 nm thickness, equivalent to two layers, considering the monolayer thickness of ∼1.03 nm. Besides TBAOH, some other organic molecules such as isopropyl amine and dimethyl sulfoxide (DMSO) have also been found to be effective intercalants for the delamination of HF-MXenes.

#### 2.1.5. Etching without Fluoride

**Figure 4:**
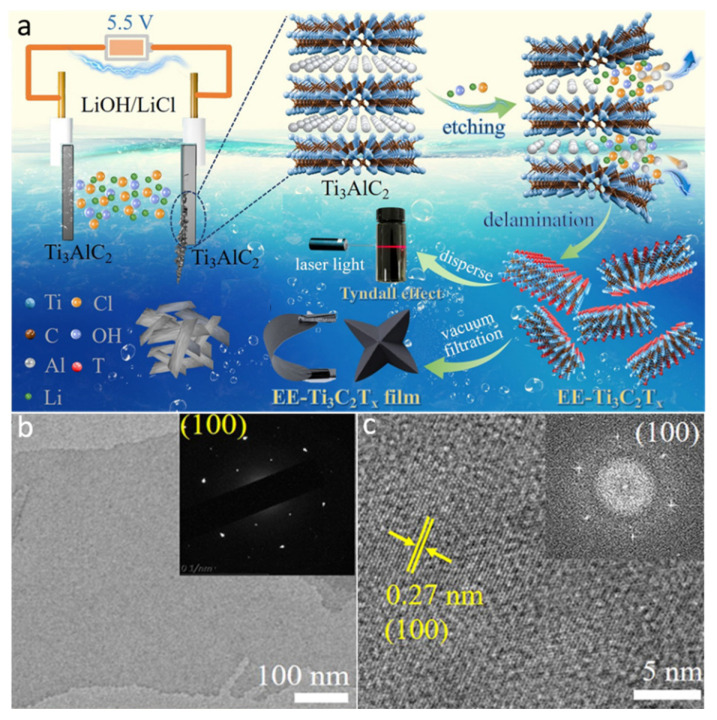
Scheme for the synthesis (**a**), TEM micrograph (**b**), and HR-TEM micrograph (**c**) of the fluoride-free Ti_3_C_2_T*_x_* MXene. Reproduced with permission from ref. [45] Copyright 2022, The American Chemical Society.

Although several etching conditions for synthesizing MXenes have been confirmed, most synthesis techniques need HF or fluoride-based chemicals, which may result in the creation of -O and -F terminations on the interface of MXene. In particular, -F terminations decrease the electrochemical performance of MXenes-based supercapacitors [43]. Thus, fluoride-free fabrication techniques are necessary to provide acceptable electrochemical performances. Li et al. [44] developed an alkali-assisted hydrothermal etching technique for the fabrication of Ti_3_C_2_ MXene using a NaOH solution as the etching agent. Because of the strong interaction between Al and alkali, alkali might be used as an etchant for Ti_3_AlC_2_ MAX phase. Obtaining high-purity multi-layered MXenes is still a big challenge. In this case, the Bayer procedure was used to etch Al layers while avoiding damage to the Ti_3_C_2_ MXene skeleton by using a high alkali concentration (i.e., 27.5 M) and a high temperature (i.e., 270 °C). Very recently, Chen et al. [45] reported the fabrication of a fluoride-free and chloride-containing Ti_3_C_2_T*_x_* MXene via electrochemical etching as revealed in Figure 4a. In the synthesis process, Ti_3_C_2_T*_x_* was delaminated via sonication in the absence of any toxic organic intercalant. The thickness of the resultant Ti_3_C_2_T*_x_* nanoflakes was ∼3.9 nm (see Figure 4b), and their dispersion in an aqueous medium was very highly stable. The SAED pattern of the Ti_3_C_2_T*_x_* (inset Figure 4b) displays hexagonal symmetry as a result of the hexagonal arrangement of the Ti-atoms at the 002 surface of Ti_3_C_2_T*_x_* MXene. The lattice fringe, with d-spacing of 0.27 nm, can be accredited to 100 planes of the Ti_3_C_2_T*_x_* MXene as is clear from the HR-TEM micrograph (Figure 4c). Based on the theoretical predictions and experimental results, the surface-attached −F remarkably hinder the electrolyte ions’ transportation and sacrifices the electrochemically active sites, which results in low performance of MXenes for applications in Li-ion batteries and supercapacitors [46,47]. Thus, the fabrication of MXenes using F-free methods is highly desirable.

### 2.2. Top-Down Approaches

For a long time, the top-down strategy has been well-established, especially in the synthesis and design of nanomaterials. These methods often involve the cleavage of 2D or 3D bulky precursor material into essential minute quantum particles [48,49,50]. Graphite, carbon nanotubes, graphene, MoS_2_ crystals, WS_2_ powder, black phosphorus, g-C_3_N_4_, and other three-dimensional and two-dimensional bulk precursors have been effectively transformed into quantum dots utilizing the top-down technique. Ball-milling [51], liquid exfoliation, chemical etching, electrochemical [52], intercalation, hydrothermal/solvothermal treatment, ultra-sonication, microwave irradiation, and other procedures are among them. The majority of the top-down approaches establish the initial O_2_-containing functionalities on the surface of catalysts, which facilitate the creation of defects in the catalysts [53]. The surface defects serve as reactive sites and allow the bulk molecules to split into tiny quantum particles [54]. This technique is of great significance because it can be operated at low temperatures. Further, copious raw materials can be used in this technique, and large-scale production can be achieved. The low yield and the need for particular treatments are, however, drawbacks. The most extensively utilized top-down approaches for the fabrication of MXene include ultrasonication, ball-milling, intercalation, hydrothermal, solvothermal, micro-explosion, and acid reflux. In this section, the details of top-down approaches for the synthesis of MXenes are discussed.

#### 2.2.1. Hydrothermal Approach

**Figure 5:**
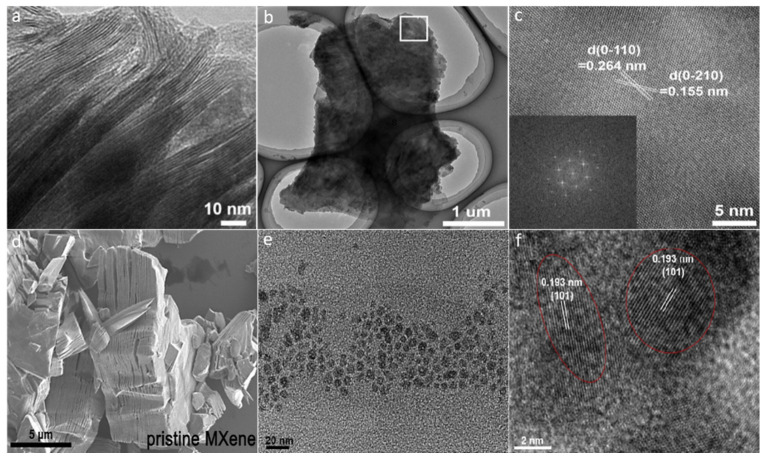
TEM images: (**a**) Side view, (**b**) bird’s-eye view, and (**c**) selected area HR-TEM with inset FFT image of *h*-Ti_3_C_2_ flakes. Reproduced with permission from ref. [62] Copyright 2018, Elsevier. (**d**) SEM micrograph of pristine MXene, (**e**) TEM micrograph, and (**f**) HR-TEM micrograph of N-MXene QDs. Reproduced with permission from ref. [64] Copyright 2020, Elsevier.

This is a heterogeneous reaction approach, which involves the heating of aqueous solutions above the boiling point of water within a high-pressure autoclave containing precursor materials. The synergetic effect of the elevated temperature and pressure and the solution pH can be employed to alter the QD size, shape, morphology, and properties [55]. In addition, the pH of the solution, reaction temperature, and time period play a vital role in the production of MXene. To synthesize MXene, a reaction temperature of 100 to 180 °C and pH of 6 to 9 are kept standard [56], because the reaction duration is influenced by the pH and temperature. Furthermore, by adjusting the conditions of the hydrothermal reaction, the material size, properties, and thickness can be altered. Xue et al. [57] employed a hydrothermal technique to create water-soluble Ti_3_C_2_ MXene and observed that by altering the hydrothermal reaction temperature to 79 °C, the properties, thickness, and size of MXene can be tailored. The MXenes obtained at 100, 120, and 150 °C resulted in particles with average diameters of 2.9, 3.7, and 6.2 nm, respectively, and average thickness of 0.99, 0.91, and 0.89 nm, showing that the monolayers make up the bulk of particles. At the time of reaction, the Ti_3_C_2_ QDs exhibits the -NH surface functionalities, and at a lower temperature (i.e., 100 °C), a new MXene structure is formed in which the d-spacing value can be confirmed. On the other hand, the MXENE formed at 120 °C exhibits a fusion structure with CTi in the core and TiO_2_ on the surface. However, because most of the Ti atoms were etched away at an elevated temperature (i.e., 150 °C), an amorphous MXENE structure was formed. According to Xiao and et al. [58], below 100 °C, the MXene structure cannot be produced. They made MXene (Ti_3_C_2_T*_x_*) by varying the reaction temperature from 60 to 80 and 100 °C. Instead of MXene, abundant nanoribbons, including a few Ti_3_C_2_T*_x_* nanoflakes, were formed at a temperature of 60 °C. By increasing the temperature to 80 °C, the shrinkage of the particles size occurred, and various-sized nanodots were formed. Ultrafine nanodots are generated and uniformly spread on the nanosheets at 100 °C. More MXenes have been synthesized by applying different hydrothermal conditions [59,60]. By adapting alternative hydrothermal conditions, MXene with doped heteroatoms have also been produced, utilizing the elements’ consequent precursors. Using Nb_2_C nanosheets precursor material and L-cysteine as a source of sulfur and nitrogen, Xu and his co-workers fabricated heteroatom-co-doped Nb_2_C MXene (S, NMXene) via the hydrothermal method at 160 °C. The particles size of MXene was between 2.6 and 4.7 nm. The fabricated S, N-MXene has a lateral size of 3.56 nm, remarkably smaller than those of the MXene (i.e., 2.4 nm) and N-MXene (i.e., 2.66 nm). The S, N-MXene had an average thickness of 1.74 nm, indicating that a monolayer was developed [61]. Recently, Peng et al. [62] fabricated 2D MXene (i.e., *h*-Ti_3_C_2_) via the hydrothermal method using low-toxicity etchants (i.e., NaBF_4_, HCl). The TEM micrograph of the resultant *h*-Ti_3_C_2_ MXene (Figure 5a) revealed a layered structural morphology. As is clear from Figure 5b, the 2D *h*-Ti_3_C_2_ nanoflakes are very thin and transparent. The selected area HR-TEM micrograph of the *h*-Ti_3_C_2_ nanoflakes (Figure 5c) revealed d-spacing of ~0.264 nm and ~0.155 nm, corresponding to the (0−110) and (0−210) planes of the Ti_3_C_2_, respectively. The FFT pattern revealed hexagonal symmetry as can be seen from the inset of Figure 5c. It is important to note that the hydrothermal etching technique not only avoids the use of HF acid but is also an efficient method for the preparation of Ti_3_C_2_ nanoflakes.

#### 2.2.2. Solvothermal Approach

The solvothermal process is simple and more efficient than the hydrothermal method and has been widely utilized to make MXene. It is similar to the hydrothermal process with the mere distinction that, instead of water, a non-aqueous organic solvent is used to make the precursor solution. Because the size, distribution, and material crystallinity can be precisely regulated as needed, the solvothermal method is regarded to be more successful and adaptable as compared to the hydrothermal method [63]. Furthermore, adjusting experimental parameters such as the reaction’s temperature, time duration, and solvent type, the desired result can be fine-tuned. Feng et al. [64] used a solvothermal technique to fabricate nitrogen-doped Ti_3_C_2_ MXene. In conclusion, the exfoliated MXene was used to make three different forms of MXene QDs with varied amine doses. To fabricate NTi_3_C_2_ QDs, di-ethylene-triamine, di-methyl-formamide (DMF), and the exfoliated MXene were mixed in equal amounts at 150 °C for 8 h. NTi_3_C_2_ QDs with an average particle size of 6.2 nm and a width of 1 nm were obtained, indicating the monolayer formation. To synthesize the QDs, the solvothermal reaction time was optimized. The particle size decreased with the increase in the solvothermal reaction time. Recently, Feng et al. [64] demonstrated the fabrication of in-situ N-doped Ti_3_C_2_ MXene QDs via the amine-assisted solvothermal method. The SEM micrograph of pristine MXene (Figure 5d) confirmed multi-layers with a thickness of approximately 5 μm. The TEM micrograph (Figure 5e) reveals a remarkable reduction in the particle size of MXene (i.e., from 600 nm to 6 nm), confirming the formation of N-MXene QDs. As is clear from the HR-TEM micrograph (Figure 5f), the resultant QDs exhibit high crystallinity with a lattice spacing of 0.193 nm (101 plane). The resultant QDs revealed diverse fluorescence quenching responses toward the detection of various metal cations. Similarly, Niu et al. [65] used the solvothermal approach to fabricate three types of Ti_3_C_2_T*_x_* QDs from the Ti_3_C_2_ MXene precursor, labelling them e-Ti_3_C_2_, f-Ti_3_C_2_, and s-Ti_3_C_2_ QDs. Their particle size varied depending on the solvent, and the average particle sizes for e-Ti_3_C_2_, f-Ti_3_C_2_, and s-Ti_3_C_2_ QDs were observed to be 2.5–0.2, 3.3–0.2, and 1.8–0.1 nm, respectively. The particles’ thickness was in the range of 1.0-2.5 nm. The particles’ thickness and lateral sizes were influenced by the polarity of the solvent, oxidation reaction, and boiling point. The disadvantage of this technique is the generation of carbon quantum dots (CQDs) from organic precursors. The undesirable CQDs have an impact on the product’s optical and catalytic capabilities [66].

#### 2.2.3. Ball-Milling Approach

**Figure 6:**
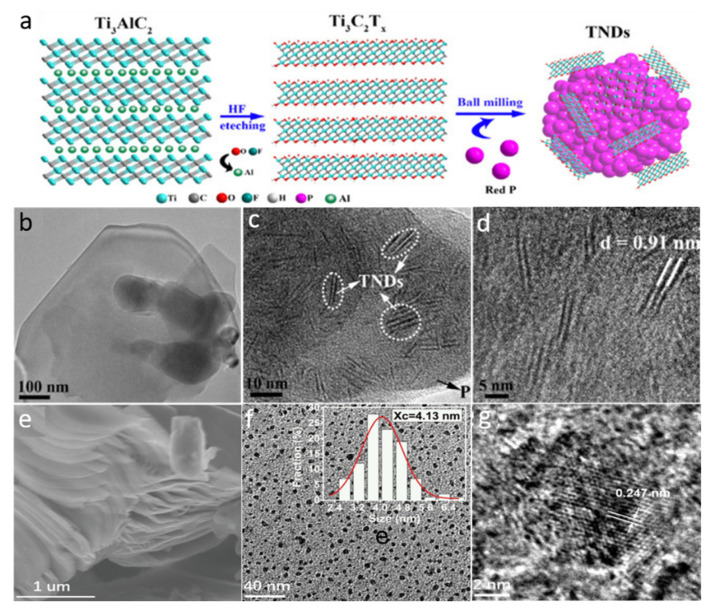
(**a**) Schematic of the synthesis process, and (**b**–**d**) FE-TEM micrographs of the Ti_3_C_2_T*_x_* nanodots. (**e**) The SEM micrograph of V2C MXene. (**f**) The average size of the generated V2C MXene was 4.13 nm, and it was 2.33 nm thick. (**g**) The lattice fringes with d-spacing of 0.247 nm. Reproduced with permission from ref. [67] Copyright 2017, The Willey.

Different QDs have been successfully fabricated via the ball-milling approach, which is an extensively used top-down method for reducing the size of nanoparticles. The physical and morphological features of the produced materials are influenced by a variety of factors such as the milling type (wet or dry), speed of the milling, the ratio of the ball to powder weight, and milling time duration. Zhang et al. [67] used the ball-milling procedure to fabricate various-sized Ti_3_C_2_T*_x_* MXene nanodots (TNDs) by mixing Ti_3_C_2_T*_x_* with diverse solid-state precursors such as red P, S, C, and Si (see Figure 6a). Ball-milling of the mixture was performed at 550 rpm for 48 h under an argon environment, while keeping the powder-to-ball weight ratio at roughly 1:20. The of FE-TEM image of the Ti_3_C_2_T*_x_* (Figure 6b) showed a μm-long thin layered structure. The red-P-assisted ball-milling of Ti_3_C_2_T*_x_* results in QDs with a lateral diameter and thickness in the range of 6–25 nm (Figure 6c). The HR-TEM micrograph of Ti_3_C_2_T*_x_* (Figure 6d) revealed an interlayer spacing of ~0.91 nm, corresponding to the (0002) planes, which confirms the development of MXene nanodots. During this process, the TiOX (X = C, P, S, Si) bond formation occurs, causing the delamination and reduction in the particle size of Ti_3_C_2_T*_x_* MXene, which is governed by the strength of the OX link. The stronger the –OX interaction, the smaller the particle size will be. Due to the strong TiOX connection, a solid-state element existed in the product. This is an ideal method used to fabricate nanocomposites.

#### 2.2.4. Ultrasonic Approach

The ultrasonic technique is an environmentally friendly and risk-free technique used for the fabrication of MXene QDs. Due to the solvents’ acoustic, cavitation, and reverberation, the ultrasonication process changes the layered, as well as nonlayered, materials into QDs. In this technique, the inherent features of materials are effectively retained. Solvents such as DMSO, DMF, N-methyl-2-pyrrolidone (NMP), and tetra-butylammonium hydroxide (TBAOH), with their high boiling points and surface energies, could overcome the binding force of bulk materials and transform them into highly dispersed, small-sized QDs. To produce the Ti_3_AlC_2_ MXene directly from the MAX phase, Yu et al. [68] used etching-assisted exfoliation and mechanical-force–assisted liquid exfoliation approaches with the solvent TBAOH. The bulk particles of Ti_3_AlC_2_ break down into smaller particles with new edges and surface sites during ultrasonic treatment. By treating the OH group with a solvent, these sites were removed as Al(OH)_4_. Meanwhile, the formation of isolated MXene sheets took place [68]. These sheets were then transformed into MXene via the bath sonication. The thickness and average lateral size of the obtained MQDs were 0.6 and 4.9 nm, respectively. Before being utilized in aqueous media via the ultrasonication technique, the organic solvents with high boiling points require extensive post-treatment. High-temperature rotary evaporation as post-treatment affects the quantum dots’ optical and catalytic capabilities. As a result, methods for synthesizing MXene in an aquatic environment gained a great deal of attention. Furthermore, widespread utilization is challenging due to the lengthy sonication method and low output yield. As a result, combining the ultrasonication technique with hydrothermal and solvothermal ones is highly effective to shorten the reaction time and increase the yield. To fabricate V_2_C MXene, Huang et al. [69] used an ultrasonication-hydrothermal method. Throughout the synthesis procedure, the V_2_C sheets were ultrasonically treated for 1 h in an Ar environment, and the suspension was treated hydrothermally at 120 °C for 6 h. The SEM micrograph of V_2_C MXene (Figure 6e) revealed a layered structure. The average size of the generated V_2_C MXene was 4.13 nm, and it was 2.33 nm thick (see Figure 6f). The lattice fringes with d-spacing of 0.247 nm (Figure 6g) were accredited to the (01¯10) plane of the V_2_C MXene. Another way to fabricate MXene is through intercalation ultrasonication. To synthesize Ti_3_C_2_ QDs, Wang et al. [70] used the ultrasonic intercalation technique. The Ti_3_C_2_ nanosheets were treated for 24 h with 25% aqueous TMAOH to produce guest intercalation and the host-layer fragment. After that, the resultant intercalated Ti_3_C_2_ nanosheets were sonicated in an Ar atmosphere for 10 h.

### 2.3. Bottom-Up Approaches

In contrast to the top-down procedures, which employ bulk material as precursors, the bottom-up approaches use molecular material as precursors. Bottom-up approaches could also be used to make MQDs from tiny precursors of organic and inorganic molecules. Bottom-up approaches have a number of advantages, including greater atomic usage, structural and morphological control, and faster functionalization, among others, which give QDs better structures and properties [71,72,73]. The previous investigations provide an excellent foundation for bottom-up MXene preparation. However, for large-scale fabrication, simple, highly efficient precursors with low toxicity, gentle reaction conditions, excellent crystallinity, monodispersity, and high yields must be addressed. Meanwhile, because of their relatively simple operating conditions compared to the top-down methods, one-pot bottom-up methodologies will likely be employed to prepare MXene in the future to fulfil the incremental application requirements. As there is a little research on the bottom-up methodologies for MQD fabrication, the study is promising, and great stress should be given to these strategies [56].

#### 2.3.1. Molten Salt Synthesis

**Figure 7:**
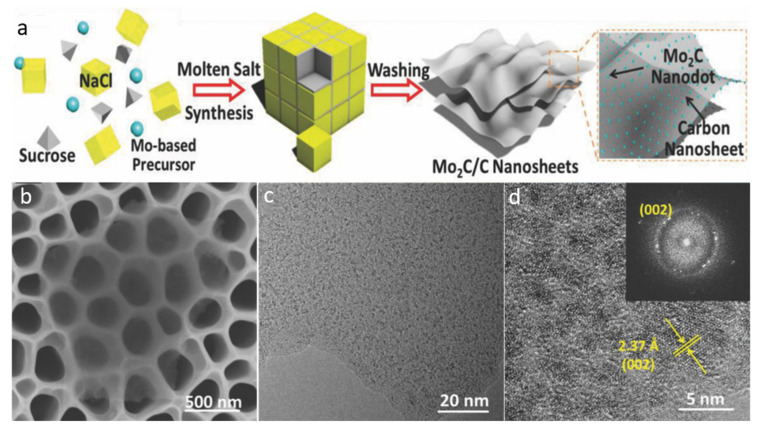
(**a**) Schematic for the fabrication of Mo_2_C/C nanosheets via molten salt method. (**b**) SEM micrograph, (**c**) TEM micrograph, and (**d**) HR-TEM micrograph with the inset showing the fast-Fourier-transform (FFT) pattern of the Mo_2_C/C nanosheets. Reproduced with permission from ref. [74] Copyright 2018, Wiley.

Due to its ease of use and short reaction time, the molten salt synthesis process has lately gained favor. Using molybdenum acetylacetonate as a precursor, Cheng et al. [74] synthesized a Mo_2_C QDs/carbon nanosheet (Mo_2_C/C) composite. In a typical synthesis, the molybdenum acetyl-acetonate precursor, NaCl, and a sucrose mixture were calcined for 2 h at 800 °C under an air environment as depicted in Figure 7a. The SEM scan corroborated the nanosheet structure (Figure 7b), demonstrating the importance of the method. The TEM micrograph further confirmed the uniform and ultrathin structural morphology of the Mo_2_C/C nanosheets (Figure 7c). According to the HR-TEM image (Figure 7d), the particle diameter implanted on the carbon sheets was 23 nm with a d-spacing value of 2.37, corresponding to the (002) plane of Mo_2_C. The nanosheet had a thickness of 3.5 nm, according to the AFM analysis.

#### 2.3.2. Pyrolysis Approach

**Figure 8:**
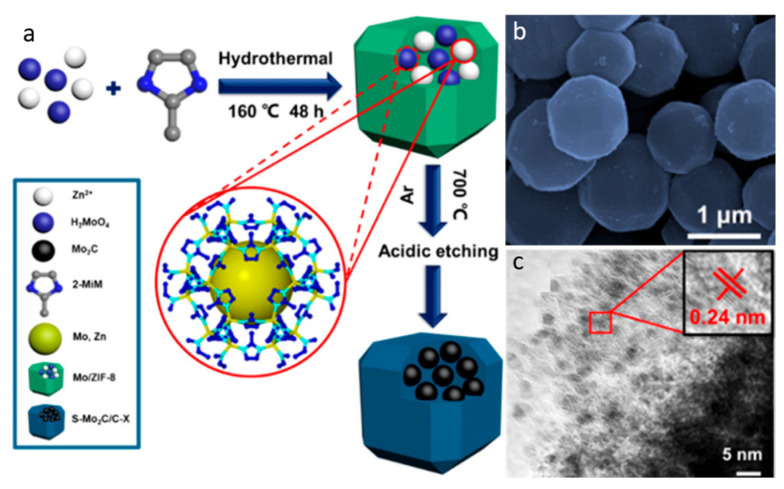
(**a**) Schematic for the fabrication of Mo_2_C-decorated carbon polyhedrons. (**b**) SEM micrograph of the Mo_2_C/C, (**c**) and TEM micrograph of Mo_2_C/C with inset the HR-TEM micrograph of Mo_2_C. Reproduced with permission from ref. [75] Copyright 2018, The American Chemical Society.

Pyrolysis is a well-known bottom-up methodology for MXene since it is a time-saving, easy, and ecologically benign procedure. Wang et al. [75] fabricated the Mo_2_C QDs-carbon polyhedron composites via a simple pyrolysis technique (see Figure 8a). In summary, the Mo/ZIF-8 was synthesized via the solvothermal method and then pyrolyzed at 700 °C for 2 h in an Ar environment before being acid etched. The SEM micrograph (Figure 8b) clearly indicated the carbonization of organic frameworks and the development of Mo_2_C as an individual polyhedron. The TEM micrograph (Figure 8c) revealed uniformly dispersed ultra-small black dots in the carbon matrix. The lattice fringe (i.e., 0.24 nm) of cubic-Mo_2_C was aligned with the (111) direction plane, as revealed in the HR-TEM image (inset Figure 8c). Despite the fact that significant works have been reported on the synthesis of MQDs using diverse synthetic techniques, further research is needed to make these bottom-up synthesis routes easy, efficient, and commercial. As a result, in bottom-up approaches, the molecular precursors with the choice of elements sources, as well as parameters of the reaction such as reaction time, temperature, and precursor concentration, are crucial in the QDs synthesis. This approach is efficient, simple, and monodisperse in comparison to the top-down ones, with high yield, improved crystallinity, and normal conditions required for the reactions. Still, very little research has been conducted on the synthesis of MXene. These approaches will receive more attention in the near future in order to address the needs of incremental applications. Carbon nanotubes have a conductivity of 15 100 S cm^−1^, whereas reduced graphene oxide has a conductivity of 15 100 S cm^−1^ [76]. As a result, the strong metallic and electrical conductivity of Ti_3_C_2_T*_x_* allowed it to work better as an electrocatalyst. In the case of surface functionalization and composition, Ti_2_CO_m_ (1.03 × 10^4^ cm^2^ V^–1^ s^–1^) has a better carrier mobility than pure graphene (0.3–1.2 × 10^3^ cm^2^ V^–1^ s^–1^) [43]. In addition, the surface functional group of Ti_3_C_2_T*_x_* (*x* = –O, –OH, –F) provides a large number of sites and influences electrocatalytic efficiency. Owing to the substantial density of state (DOS) around the Fermi level, they show strong electrocatalytic activity [43].

### 2.4. Challenges in MXenes Synthesis

The quality of MXenes is related to the synthesis procedures. In an ideal synthesis technique, the MAX phase is the precursor material, and its quality governs the MXenes product. Thus, choosing a high-purity MAX phase without unreacted substrates is vital. Further, it is essential to prepare the MAX phase with negligible impurities originating from mechanical milling. Yet, the additives can be detached as residue during delamination and etching. Additionally, if these are acid soluble, then they easily wash out through rinsing.

During MXenes etching, the MAX phase undergoes severe conditions to solubilize and remove the inclosing aluminum layers. In early methods, poisonous HF was utilized, and the most substantial task was associated with the researcher’s safety. Later, based on optimal conditions and less-toxic etchants, the MILD technique was developed. Nevertheless, the method was still based on the utilization of HF obtained from the LiF and HCl mixture. More innovation, by utilizing ionic liquids or molten-salts, allows for safe and less hazardous synthesis routes of MXenes. Yet, their upscaling is extremely restricted owing to the expensive chemicals.

The oxidation of MXenes is crucial, and it is important to adjust features of the MAX phase and the processing time. Thus, the more serious feature of the MAX phase is their grain sizes. For example, a MAX phase with large size grains produces unetched MXenes, which settle in the colloidal solution and require a second step of etching to remove Al entirely. On the other hand, a MAX phase with small grains size etches very easily but needs a long milling time and results in additional impurities, which come from the ball milling and vessel. Furthermore, a MAX phase with a small grain size produces small flakes of MXenes. Thus, a MAX phase with a small grain size results in low-quality MXenes.

A comparable deliberation can also be made for the delamination method. For example, MXenes with a large grain size will not delaminate entirely and requires a longer time for delamination. Further, if the delaminated flakes are not reduced via sonication, they would have large dimensions. From this perspective, the adjustment of the materials’ parameters is a challenging task as the fabrication methods produce diverse results in terms of the flakes’ parameters.

To characterize the as-prepared MXenes, several parameters including the surface-zeta potential, conductivity, and Raman and XRD analyses should be carefully considered. For examples, different MXenes functionalities would give similar Raman spectra and XRD patterns because these techniques are precise to the materials’ composition and physical nature of the bulk. However, MXenes nanosheets might differ in their physical nature. Thus, it is a challenging task to regulate and track changes in the chemistry and dimension of flakes (surface functional-group arrangement) via cutting-edge and complementary materials characterizations.

The elimination of Al etching by-products is also a crucial step and vanishes when the solution pH reaches 6 or 7. If the concentration of the flake’s dispersion is high, a substantial quantity of the adsorbed inorganic Li^+^ or Cl^–^ ions or organic TMA^+^ ions might be difficult to wash out, and several washing, centrifugation, or filtration steps are required to attain the desired pH.

MXenes types and their stoichiometry also effect their synthesis. For example, etching and delamination of Ti_3_C_2_T*_x_* MXene in LiF/HCl is an easy process. On the other hand, the Nb_2_CT*_x_* MXene can firstly be etched with HF and further delaminated with TBAOH. This is because the LiF/HCl method is less effective for such types of MXenes. Thus, Ti_3_C_2_T*_x_* fabrication could be up-scaled. It is difficult to etch and delaminate several types of MXenes, such as Cr_2_CT*_x_*, V_2_CT*_x_*, and Ti_2_NT*_x_*, into single flakes because of their high oxidation tendency and low stability. Thus, the resultant MXenes would be multi-layered in these phases.

The problem related to the structural preservation of MXenes is due to their oxidation stability. MAX phases exhibit high stability due to the existence of interleaving “A” layers and eliminating this layer via etching can markedly decrease the multi-layered structures’ stability. Nevertheless, the multi-layers still exhibit enough stability to endure the preparation of samples for different analysis and industrial applications. In addition, the MXenes structures are destabilized with delamination to a single or few layers, and after several hours, these layers oxidize under ambient conditions and further decompose after a few months. Subsequently, the materials should be kept in a dark and oxygen-free atmosphere at a low temperature.

The above issues also effect the characterization of the materials. For example, the materials will be immediately characterized after fabrication because of the rapid oxidation. The majority of the techniques operate under ambient environments, (i.e., in air, or water presence), and materials are oxidized during preparation for characterization. These changes may be pursued by varying the XRD and Raman patterns. Thus, to avoid the oxidation of MXenes, the appropriate laser intensity for Raman analysis should be chosen. Regarding the surface chemistry, surface changes can be evaluated via the zeta-potential or XPS techniques. By measuring the sample immediately after fabrication, its zeta-potential value would be extremely negative. The immediate XPS analysis after fabrication would detect the metallic bonding with hydroxide groups and fluorine without any metal oxide. Upon oxidation of the sample, the zeta-potential would be close to zero and the XPS would confirm the existence of metal oxides [77,78,79,80].

## 3. Properties of MXenes

Because of the abundance of electrons associated with transition metal atoms, MXenes have a broad and changeable surface interaction. The fascinating properties of MXenes, including the physical, chemical, magnetic, mechanical, electrical, surface, thermal, as well redox, are controlled by the MAX phase, etching and raw materials, and delamination and etching strategies. Furthermore, the MXenes properties can be modified for appropriate applications by changing its composition, adopting alternative elements of “M” and “X”, and altering the surface terminal functionalities. Recently, theoretical analyses utilizing computer approaches have characterized the features of MXenes. The most attractive qualities linked to energy storage are discussed below [81].

### 3.1. Structural Properties

Due to their exceptional conductivity and abundant surface functionalities, MXenes with high structural and chemical stability can act as both carriers of inherent active and/or other functional materials for various applications including energy conversion and storage [82]. MXenes, on the other hand, have the drawbacks of ultrathin 2D materials [83], namely a significant tendency to restack and the lack of a restricted porosity structure. Over the last three years, many efforts have been devoted to designing and modifying porous MXenes. Until now, a variety of porous MXenes with acceptable topologies have been fabricated using a variety of synthetic techniques and used in a wide range of applications, of which all revealed enhanced performances. The goal of Fanxing Bu pioneer is to bring a new understanding to the relationship between the fundamental synthesis, composition, structure, and performance of porous MXenes, which will spur further progress and serve as a strong platform for practical applications [15]. Due to their beneficial structures, 2D porous MXenes have also been utilized for various applications, inspired by their porous structures and tunable physicochemical properties. Table 1 explains, in detail, the role of porous structures, as well as the particular functions of pores in the associated applications [15].

### 3.2. Optical Properties

The optical features of Ti_3_C_2_T*_x_* MXenes include high transparency and a photothermal effect. Ti_3_C_2_T*_x_* films have a strong surface plasmonic effect, which is an important feature. Ying et al. [84] achieved transparency of more than 75% in the visible and near-infrared ranges, despite the fact that transparency decreases as the spin rate of the coating decreases. The generated film is very transparent at 550 nm, according to a V_2_CT_X_ transmittance investigation, and the transmittance decreases as the film thickness increases. They attained 89% maximum transmittance for an 11-nm-thick layer. The absorbance coefficient of the film was 1.22 ± 0.05 × 10^5^/cm at 550 nm. This value is lower than those of the Ti_2_CT*_x_* (i.e., 2.7 ± 0.1 × 10^5^/cm) and Ti_3_C_2_T*_x_* (i.e., 2.4 ± 0.1 × 10^5^/cm) MXenes. According to this investigation, the annealing temperature of the MXenes film has no effect on the transmittance or absorption coefficient. This is in direct opposition to the conductivity findings. Zhang et al. [85] made 2D titanium carbide MXene with a transparency of >90%. The synthesis method involves an irreversible color shift that occurred during the Ti_3_C_3_T_Z_ intercalation stage. However, there has been little research on the effect of synthesis on optical characteristics [86].

### 3.3. Thermal and Electrical Properties

Thermal conductivity and thermal oxidation are both high in Ti_3_AlC_2_. The thermal conductivity of the Ti_3_C_2_F*_x_* monolayer is approximately 108 W/(mK), while the Ti_3_C_2_O*_x_* monolayer is approximately 11 W/m K [87]. However, with Ti_3_C_2_T*_x_* films, it is found to be approximately 2.84 W/(mK) in experiments. The metallic conductivity of Ti_3_C_2_T*_x_* sheets is 2400 S cm^−1^ and is extremely flexible. V_2_C is a kind of MXene that has been investigated and found to have a conductivity of 3300 S cm^−1^. Its versatility makes it perfect for applications such as wearable electronics [88]. The thickness of the layers and calcination temperature have a great effect on the sheet conductivities. According to Ying et al. [84], after annealing, the conductivity increased, and the sheet resistance for thicker films was lower than for thinner films.

### 3.4. Mechanical Properties

The mechanical characteristics of MXenes can vary significantly based on the surface terminations. Magnuson et al. [89] discovered that the surface terminal groups weaken the Ti–C bond by removing the charge from them. They discovered that the Ti-C bond in Ti_2_C-T*_x_* is longer than in Ti_3_C_2_-T*_x_*, which might affect the elastic properties of the materials. The scientists also proposed that modifying the binding strength could help to improve flexibility. Because of their higher mechanical strength, Zha et al. [90] indicated that MXenes with an O-terminal functionality should be the first choice for structure materials, supercapacitors, and other applications.

MXenes have one distinctive characteristic, i.e., their excellent mechanical strength. Despite the significant improvements in the fabrication of MXene nanostructures, Ti_3_C_2_T*_x_* has been extensively explored for a variety of applications owing to its exceptional conductivity, mechanical capabilities, and high electro-magnetic shielding effect [91]. MXenes revealed high mechanical ion adsorption capabilities in numerous investigations, paving the path for more research into their possible usage in sensors and flexible electronics [92]. The MXenes’ mechanical properties with composition variation are summarized in Table 2 [93]. In the current 5G era, electronic gadgets on the market are growing at an exponential rate, resulting in significant electromagnetic pollution that endangers human health and device operation. As a result, lightweight EMI-shielding materials with exceptional mechanical strength and a high shielding efficiency (SE) have received huge attention. Due to their hydrophilic nature, exceptional specific surface area, and superior conductivity, 2D transition metal carbides and carbonitrides achieved excellent performance in the field of EMI-shielding. In one study, Ti_3_C_2_T*_x_* MXene was mixed with a formaldehyde and resorcinol solution in a sol-gel technique to generate a hybrid gel and Ti_3_C_2_T*_x_* MXene/C hybrid-foam (MCF) via freeze-drying and heat reduction. The vacuum-assisted impregnation and curing methods were utilized to make MCF/epoxy EMI-shielding composites. The conductivity, EMI performance, and mechanical strength of the MCF/epoxy EMI-shielding composites were thoroughly investigated. The structure of the resulting MCF was investigated using Raman, XPS, and SEM. On this premise, the impact of the Ti_3_C_2_T*_x_* MXene mass fraction on the electrical conductivity, EMI performance, and mechanical strength of the MCF/epoxy EMI-shielding composites was studied in depth [94].

## 4. Applications of MXenes

MXenes gained tremendous research interest due to their unique properties, including their superior electrical conductivity [76], high volumetric-electrochemical capacitance [95], tunable plasmonic features and work function [95], optical transparency and electrochromic in thin films [96], high thermal stability [97], good mechanical strength [98], and their capability to form highly stable colloidal solutions in various polar solvents, especially alcohols and water [95]. MXenes have been utilized in a variety of fields, such as energy storage, electronics, structural components, and environmental cleanup, due to their attractive qualities. Energy storage was the first application of MXenes and is the most wide-ranging to date [99], but it has since been extended to include supercapacitors and all types of batteries [99,100,101]. Even in clear 40–50 nm layers, MXenes showed higher efficiency than graphene and carbon-based composite materials and were found to be effective as metals in communication [102] and electromagnetic interference (EMI) screening [103]. The removal of heavy metals, desalination, and the adsorption of pollutants are all examples of how MXenes are being employed in environmental remediation [104,105]. Other potential applications of MXenes include catalysis, electronics, and biological applications such as photo-dynamic cancer therapy [105], dialysate-regeneration [87], implanted electrodes [106], and intraocular-lenses [107]. In medical technology, when MXene films substitute an Au or Pt metal electrode, it not only enhances the performance but also saves money. In terms of their applications, the industry should focus on the use of Mxenes [95].

### 4.1. Photocatalysis

**Figure 9:**
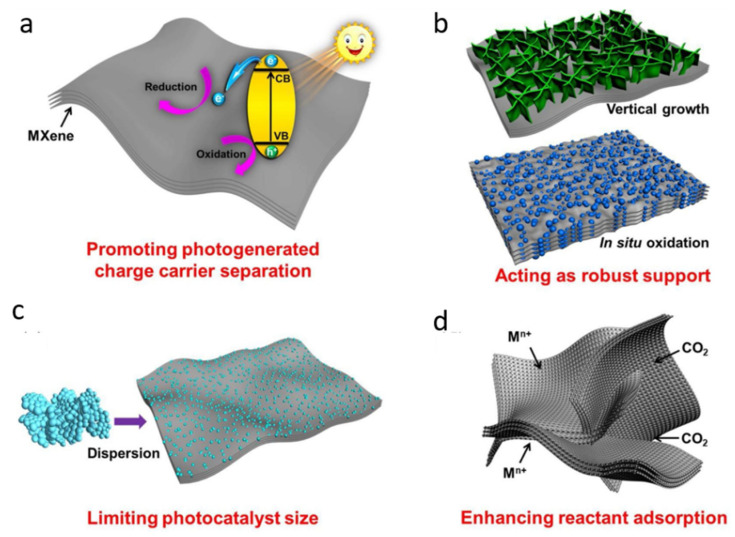
(**a**–**d**) Layered MXenes and their roles in photocatalysis. Reproduced with permission from ref. [118] Copyright 2020, Elsevier.

Photocatalysis is a catalytic approach used for solar energy conversion into chemical fuels and the decontamination of various pollutants from the environment [108,109,110,111,112,113,114,115,116,117]. The process occurs in various steps: (i) Charge carrier’s generation, (ii) the separation and migration of photogenerated charge carriers to the surface of the photocatalyst, and (iii) redox reactions via the aid of photoinduced electrons and holes (see Figure 9a–d) [118]. The charge carrier’s separation and transfer is regarded as the rate-determining step in photocatalysis because rapid charge carrier’s recombination greatly reduces the photoactivity and solar-to-chemical energy conversion efficiency of the catalysts [119]. In light of these exciting qualities, the design and fabrication of novel 2D materials with unique optoelectronic structures has received tremendous interest in photocatalysis.

MXene (transition metal carbide, nitride, and carbonitride), a novel class of 2D materials, was first reported in 2011 and has steadily grown in popularity as a photocatalytic material [120]. MXene was initially investigated for solar fuel conversion and storage devices (i.e., supercapacitors and batteries) owing to its large theoretical gravimetric capacitance and super conductivity [30,121,122]. Since 2014, MXene has been intensively investigated in photocatalysis, with a significant increase in literature based on MXene-based photocatalysts. The following factors are mostly responsible for MXenes’ effective photocatalysis applications: (i) The abundance of functional groups produced by the wet chemical etching technique is advantageous for making a close-contact boundary between MXene and the coupled semiconducting material, (ii) the MXene surface chemistry can be tweaked to change the bandgap alignment, and (iii) the highly conductive metal centers of MXene’s multilayer structure improve its metallic conductivity and electron-accepting capabilities [123]. As a result, MXene is seen as a viable alternative to other reported 2D materials, and it has been extensively investigated in photocatalysis for various applications such as CO_2_ conversion, water splitting, pollutant oxidation, and N_2_ fixation. MXene could improve photocatalytic activity in these applications by performing a variety of functions, including boosting charge carrier’s separation and transfer, acting as a strong support, restricting photocatalyst size, and promoting adsorption of reactants [118].

As a new class of layered material, MXene has a variety of unique photochemical features and infancy variables that must be further researched and investigated in order to produce useful photocatalytic effects. Such advantages of MXene-based composites could lead to the enhancement in photocatalysis for practical applications. As a result, a thorough examination of MXene could provide useful insight into the development of MXene-based heterostructure materials. This perception analyzes the underlying role of MXene in promoting photoactivity and presents the recent progress made in the design of MXene-based heterostructure photocatalysts [124].

An effective synergetic interface could improve photocatalytic reaction processes and could greatly affect the performance of 2D/2D MXene-based heterostructure photocatalysts due to the noticeable “face-to-face” interactive contact and different interface areas’ exposure [125]. In fact, the two-dimensional MXene structure can provide a large number of surface groups for creating 2D/2D heterojunctions with other two-dimensional photocatalysts. Close “face to face” contact in a 2D/2D MXene-based heterojunction offers a great deal of potential for increasing photo-induced charge carrier separation and transfer across the heterojunction interface. Photo-induced electrons triggered by the 2D heterostructure catalysts could aggregate onto the surface via electron transfer, thanks to the strong photocurrent and outstanding electronic conductivity of MXene’s. The 2D structural platform of MXene can act as an electron mediator, enabling the fast extraction of electrons from 2D materials for better photocatalytic activities [124]. Photocatalytic activities of MXene-based composites are summarized in Table 3.

### 4.2. Electrocatalysis

#### 4.2.1. MXenes-Based Electrocatalysts for HER

Hydrogen energy development provides a cost-effective response to the current environmental and energy problems. In the proposed hydrogen economy, it is necessary to design HER (hydrogen evolution reaction) catalysts with excellent performance, such as high stability, superior conductivity, and selectivity [136]. The electrocatalysts with high performance can reduce the required overpotential for HER, thereby increasing the efficiency [137]. The hydrogen adsorption Gibbs free energy (GH), which is regarded to be the descriptor of HER activity as a first approximation [138], can be calculated using density functional theory (DFT). The best HER activity is achieved when the GH is close to thermal-neutral. MXenes has better potential than other NPM-based HER electrocatalysts and exhibit excellent physicochemical properties. For example, (a) MXene surfaces contain a large number of –O and –OH groups, which can form strong bonds with other semiconductors surfaces. Furthermore, (b) the super electrical conductivity of MXenes allows for efficient charge-carrier transport. Additionally, (c) MXenes have abundant exposed metal sites at their terminals, which allow them to reveal higher redox activity than carbon compounds. (d) MXenes also exhibit high hydrophilicity, which guarantees they make appropriate contact with water molecules. Lastly, (e) MXenes exhibit exceptional chemical and structural stability in aqueous media. In addition, HER electrocatalysts based on MXenes received tremendous attention in recent years, and a great deal of experimental and theoretical work has been performed. Through structural engineering, MXenes have been optimized intrinsically and extrinsically in terms of terminal groups’ modification, metal doping, hybridization, and nanoarchitecture. As HER electrocatalysts, MXenes and their composites show great potential in substituting Pt-based catalysts (Table 4) [139].

#### 4.2.2. Termination Modification

**Figure 10:**
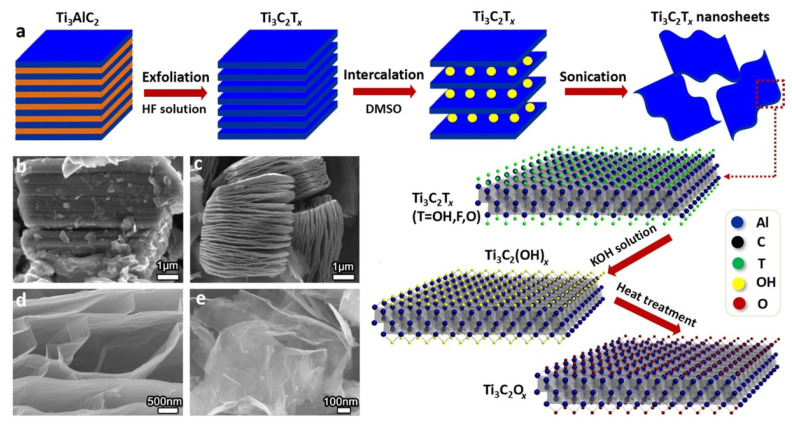
(**a**) Schematic for the fabrication of Ti_3_C_2_T*_x_* MXene and E-Ti_3_C_2_O*_x_*. SEM micrographs (**b**) of Ti_3_AlC_2_, (**c**) layered Ti_3_C_2_T*_x_*, (**d**) layered Ti_3_C_2_T*_x_* intercalated with DMSO, and (**e**) E-Ti_3_C_2_T*_x_*. Reproduced with permission from ref. [141] Copyright 2019, Willey.

MXene termination modification is thought to help in the progress of HER performance by increasing the conductivity and surface termination. The termination modification of MXenes is widely known for optimizing the electronic structure and constitutionally promoting the HER activity. Thus, MXene termination modification has been studied extensively in recent years, both experimentally and theoretically [130]. Experimentally, the 2D MXenes containing O/OH terminal groups displayed metallic characteristics and indicated good charge transfer. Surface Pourbaix diagrams confirmed the experimental findings, demonstrating that this type of MXenes exhibits high surface chemical stability, resulting in a significantly higher exchange current and enhanced hydrogen evolution. The GH was determined, indicating that their surface terminal oxygen atoms serve as active sites for HER. The contact between 2D MXenes and H was aided by these surface oxygen atoms. Further research revealed that the Heyrovsky mechanism was involved in the HER process over these MXenes. MXenes with various surface functionalities (i.e., –OH, –O, –F) were used to study their HER activity. The O-terminated surface MXenes revealed excellent performance compared to those with OH– and F-terminated surfaces [140]. There have been a number of other research studies on termination modification. Based on the ultrathin O-functionalized Ti_3_C_2_ MXenes, Jiang et al. [141] developed an effective HER catalyst as depicted in Figure 10a–e. On the basal plane, the equivalent F-functionalized MXenes were harmful to HER, slowing the kinetics of hydrogen adsorption. Ti_3_C_2_O*_x_* MXene was made by scattering Ti_3_C_2_T*_x_* in an aqueous KOH electrolyte and reducing the F-termination with the OH ones. The resultant Ti_3_C_2_(OH)*_x_* was then annealed at 450°C in an Ar environment by converting the OH groups to O-terminal ones via the dehydration reaction. The Ti_3_C_2_O*_x_* nanosheets were regarded as an excellent HER electrocatalyst. It had a 190 mV overpotential at 10 mA cm^2^, significantly lower than those of Ti_3_C_2_(OH)*_x_* (217 mV) and Ti_3_C_2_T*_x_*-450 (266 mV). The extremely active O-sites on the Ti_3_C_2_O*_x_* basal plane resulted in a 60.7 mV dec^−1^ Tafel slope. This research establishes the groundwork for altering the surface terminal groups of MXenes-based HER electrocatalysts to improve their performance. In short, the functionalities on the basal plane regulate the HER performance of MXenes. The higher concentration of F on the basal plane showed reduced HER activity for Ti_3_C_2_, Mo_2_C, and Mo_2_Ti_2_C_3_ Mxenes [142]. For HER, the O-terminated Ti_3_C_2_ MXene is a suitable electrocatalyst. MXenes with other surface functionalities (i.e., –Cl, –S, and –Br) have been synthesized in the past, but their HER performance was not studied [143].

### 4.3. Nitrogen Fixation

**Figure 11:**
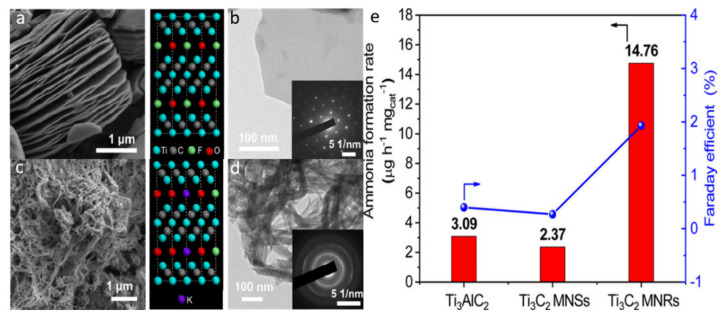
(**a**) SEM micrograph along with structural model, and (**b**) top view of TEM micrograph with the SAED pattern of the Ti_3_C_2_ MNSs in the inset. (**c**) SEM micrograph along with structural model, and (**d**) TEM micrograph with SAED pattern of the Ti_3_C_2_ MNRs in the inset. (**e**) Formation of ammonia and the faradaic efficiency with various electrodes at −0.5 V potential versus the RHE after electrocatalytic tests for 3 h at room temperature. Reproduced with permission from ref. [148] Copyright 2020, Willey.

Under mild circumstances, the synthesis of ammonia (i.e., N_2_ + 3H_2_ → 2NH_3_) is an interesting and attractive catalytic reaction [144]. Fritz Haber and Carl Bosch discovered and implemented the catalytic conversion of N_2_ to NH_3_ at large-scale, currently known as the Haber Bosch (HB) process. This process uses multi-promoted fused Fe catalysts operating at elevated temperatures (i.e., 400–500 °C) and pressures (i.e., 100–300 bar) for NH_3_ synthesis [145]. Because of the chemical inertness of N_2_, the severe circumstances require large, yet essential, energy expenditure. In reality, the HB rate-limiting step is kinetically obstructed dissociation. As a result, establishing an efficient and practically applicable N_2_ fixation technique is critical. For instance, Carbide and Nitride MXenes with 2:1 stoichiometry revealed exceptional N_2_ activation capabilities, with substantially exothermic adsorption energies [146]. MXenes have been regarded as emerging catalysts for low-pressure (i.e., 1 bar) N_2_ dissociation, based on the first-principle density functional calculations and micro kinetic simulation studies. Their activation barrier for N_2_ dissociation is actually inferior to that of Ru nanoparticles. MXenes can adsorb N_2_ exothermically with relatively high energy of adsorption (i.e., 1.11–3.45 eV) and 20% extension of the N_2_ bonding length. Compared to the crystal surfaces of Ru (0001), which have energy barriers in the range of 0.4–2.0 eV due to their active sites, this substantially simplifies the dissociation, with an energy barrier less than 1 eV. MXene (W_2_N) is projected to be the most appropriate electrocatalyst with the smallest energy barrier (i.e., 0.3 eV), and can be easily managed under ordinary pressure and temperature conditions [147]. For instance, Wei et al. [148] reported the fabrication of Ti_3_C_2_ MXene nanosheets, which were then transformed into Ti_3_C_2_ nanoribbons. The material was employed in N_2_ fixation for ammonia synthesis. The SEM micrograph (Figure 11a) of the resultant Ti_3_C_2_T*_x_* (T = F, OH) after etching with HF revealed a 2D layered structure, demonstrating the effective exfoliation of Al from the Ti_3_C_2_Al MAX phase. The TEM micrograph (Figure 11b) clearly revealed a sheet-like structure with a single-layer nanosheet. From the SAED pattern (inset Figure 11b), it was confirmed that the resultant MXene has a monocrystalline nature. After treating the Ti_3_C_2_ nanosheets with KOH, the Ti_3_C_2_ nanoribbons were formed as clear from the SEM micrograph (Figure 11c). Further, it was confirmed by the HR-TEM micrograph (Figure 11d) that the Ti_3_C_2_ nanoribbons exhibit a diameter of approximately 16 nm. The 3D structure of Ti_3_C_2_ MXene nanoribbons led to the significantly enhanced N_2_ fixation performance due to the surface-exposed Ti−OH sites (Figure 11e). Based on the above discussion, it is confirmed that MXenes have promising applications in N_2_ fixation.

### 4.4. Gas Sensing

**Figure 12:**
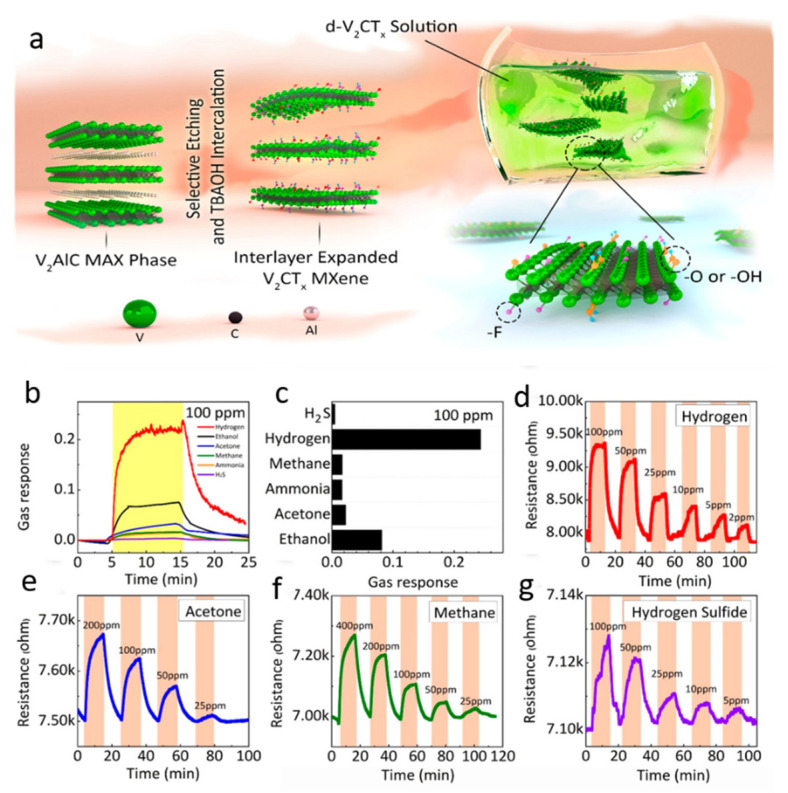
(**a**) Schematic for the delamination of V_2_CT*_x_* MXene. (**b**) The compiled resistance variation of the V_2_CT*_x_* gas sensor toward 100 ppm gases at room temperature. (**c**) Sensor response toward 100 ppm of hydrogen sulfide, hydrogen, methane, ammonia, acetone, and ethanol at room temperature. Real-time sensing response of the V_2_CT*_x_* sensor toward varying concentrations of different gases: (**d**) Hydrogen, (**e**) acetone, (**f**) methane, and (**g**) H_2_S. Reproduced with permission from ref. [156] Copyright 2019, the American Chemical Society.

Gas sensing is an emerging research direction because it is employed in air quality control for monitoring pollution, breath analysis, and diagnosis [149,150]. Aside from highly reactive gases such as NO_2_ and H_2_S, the detection of volatile organic compounds (VOCs) at parts per million (ppm) concentrations is crucial for the early identification of stern disorders such as peptic ulcers. Usually, the low electrical noise and high-signal throughput are the two critical problems in developing a sensitive gas sensor [151]. This is likely in the case that the sensing platform can accommodate both the high conduction and abundant active sites for interfacial interactions. A trade-off relationship occurs in traditional materials, such as graphene, metal oxides, and black phosphorous (PB), which classifies them as appropriate but not optimal for sensing applications [152,153]. MXenes provide numerous advantages in gas sensing due to their highly conductive surfaces with appropriate dangling functions and adsorption active sites [154]. MXene (Ti_3_C_2_T*_x_*) has been widely researched as a substrate for detecting various gases including acetone (CH_3_COCH_3_), ethanol (C_2_H_5_OH), ammonia (NH_3_), propanol (C_2_H_5_CHO), and some highly reactive and hazardous gases such as NO_2_, SO_2_, and CO_2_. The gas response was detected in terms of the change in the resistance of the gas adsorption substrate (i.e., Ti_3_C_2_T*_x_*), standardized against the N_2_ gas resistance baseline. Unlike conventional materials, Ti_3_C_2_T*_x_* shows a positive change in resistance for all gases, independent of their composition. This indicates that MXene exhibits the universal capability of the adsorption of gases. The molecules of gases interact with the surface functionalities of Ti_3_C_2_T*_x_*, altering its resistance and lowering its conductivity. In addition, the maximum responses toward ammonia, propanal, acetone, and ethanol were observed to be 0.8%, 0.88%, 0.97%, and 1.7%, respectively, with lower responses for SO_2_, NO_2_, and CO_2_. Further, the sensitivity response of the sensor was investigated to clarify whether it varies with MXene sheets thickness. The DFT results revealed that the metal-like conductivity and high energy of adsorption of the surface terminal moieties are responsible for the sensor’s sensitive response. This was intriguing since it implied that Ti_3_C_2_T*_x_*’s unique functionalities may be utilized to enable selective gas sensing. W. Yuan and his co-worker converted MXene into a 3D polymer network to improve its sensitivity for VOCs such as ethanol, methanol, and acetone. An electrospinning method was employed to form a 3D network, which involved the mixing of aqueous electrolytes of the positively charge polymers (PVA/PEI) and the negatively charged MXene (Ti_3_C_2_T*_x_*). The electrostatic interaction allowed the MXene nano-flakes to self-assemble over the polymer network via the electrospinning process and resulted in the development of a 3D porous fibrous network. This interconnected porous nature of the network allowed the diffusion of large molecules of gas and VOCs detection, even at low concentrations of 0.1–0.17 ppm, with detection up to 50 ppb. Further, the sensing ability of the 3D porous architecture was compared with that of the bare MXene, which was exposed to 5 ppm of the VOC gases, and its signal response was twice that of the bare Ti_3_C_2_T*_x_*. This reveals the potential significance of the 3D network [155]. In another report, MXene (V_2_CT*_x_*) was used to construct a sensor via a more contemporary technique. Though the majority of early work on gas sensors was based on Ti_3_C_2_T*_x_*, this study revealed that MXenes with abundant compositions can be employed as promising materials for gas sensing applications. De-laminated V_2_CT*_x_* sheets (Figure 12a) were used to make an inter-digitated platinum electrode that could detect polar and non-polar gases such as H_2_ and CH_4_. In addition, the responses for ethanol, acetone, NH_3_, CH_4_, H_2_, and H_2_S were 0.0816, 0.0226, 0.0166, 0.0167, 0.2435, and 0.005 (Figure 12b,c). Further, the real-time sensing response of the V_2_CT*_x_* sensor towards varying concentrations of different gases, including (d) hydrogen, (e) acetone, (f) methane, and (g) H_2_S, is depicted in Figure 12d–g. MXenes based on vanadium exhibit high sensitivity towards H_2_ gas rather than the Ti_3_C_2_T*_x_* MXene (highly sensitive towards NH_3_). Thus, altering the mixture of MXene materials could form different interfacial contacts, affecting the selectivity toward various gases. Another technique was to investigate the interlayer swelling of Ti_3_C_2_T*_x_* to clarify how it effects the capability of gas sensing [156]. The gas sensing performance of MXene-based composites is summarized in Table 5.

### 4.5. Supercapacitors

Due to their exceptional metallic conductivity, MXenes are frequently used in super-capacitor applications to assure fast electron transport; particularly, Ti_3_C_2_T*_x_* is the most promising candidate. It was discovered that metal ions can spontaneously or electrochemically intercalate 2D MXenes, indicating that MXene has the capacity to store energy [164]. The ions span the shallow and deep adsorption sites in MXenes and could be accommodated initially at low adsorption sites along the particle’s edges, and subsequently, at the deeper sites in the particle’s interior, which exhibit high activation energy of the ion adsorption. As a result, the ions may be adsorbed quickly on the shallower absorption sites at high scan speeds to confirm an exact volume of charge storage. This makes MXenes the most attractive choice with increased rate performance. Further, the cation intercalation may affect the fluctuation of the water confined between layers. The nanosheets of MXenes are interpolated with kosmotropic ions (i.e., Mg_2_^+^, Li^+^, and Al_3_^+^) particularly in the partially hydrated form without any dehydration, whereas the chaotropi -ions (i.e., TEA^+^ and Cs^+^) effectively dehydrate the Mxenes [165]. During cycling, fluctuations in c-values are frequently caused by the ions’ extraction from and insertion into aqueous media. In contrast to the interlayer gap extension in graphite, different ions in MXene cause different deformation behaviors. If the interpolated cations exhibit higher charges and a short ionic radius, the interlayer gap between the Ti_3_C_2_T*_x_* MXene nanosheets will be reduced, whereas cations with low charges and a large ionic radius would result in the extension of interlayers in MXene.

#### 4.5.1. Nonaqueous Systems

As mentioned earlier, the hydrated-cations intercalation in electrodes of MXene occurs the in aqueous media. Inside the insignificant potential window of aqueous media, an electric double-layer is formed by the hydrated cations in the interlayer section, which results in a distinctive capacitance performance. Nevertheless, the solvated cations are interpolated into the space region of interlayers in the primary phase of the charging cycle. While employing the non-aqueous electrolytes, collapse of the solvation shell occurs because of the significant difference in the inner potential of the interlayer space region. Thus, the desolvated ions entirely interpolate upon successive charging, and overlapping of their atomic orbitals occurs with MXenes orbitals to form a donor band. The expansion of this donor band reduces MXenes and results in intercalation pseudo-capacitance owing to the transfer of charges from these ions to the MXene sheets [166].

When compared to the aqueous environments, the electrochemical behavior of Ti_3_C_2_T*_x_* MXene in nonaqueous fluids is radically different. Nonaqueous environments such as organic electrolytes and ionic liquids have been extensively studied due to the broad potential windows, resulting in high energy densities. For instance, Lin et al. [167] investigated the Ti_3_C_2_T*_x_* MXene electrochemistry in an ionic-liquid electrolyte (i.e., EMITFSI in acetonitrile). At 0.2 V and 0.6 V, the cyclic voltammograms displayed large peaks. In fact, MXene with these terminal groups is electrochemically inactive in ionic-liquid electrolytes, and the pseudo-capacitance features are not expected to occur in aqueous systems as compared to the Ti_3_C_2_T*_x_* Mxene [88]. These peaks result from the intercalation of the EMI^+^ and TFSI ions in Ti_3_C_2_T*_x_* MXene in both negative and positive voltage windows. At the Ti_3_C_2_T*_x_*/electrolyte (LiPF_6_/EC-DMC) interface, partial desolvation of Li^+^ ions are seen, following the charge storage (redox reactions) after intercalation. These redox reactions take place over a widespread range of voltage values (i.e., 0.05 V to 3 V). It happens in a row, resulting in a series of large redox peaks related to the intercalation of Li^+^ ions.

#### 4.5.2. MXenes-Composites

Approximately 30 different types of MXene have been reported, and several types have been utilized for applications in supercapacitors. Many considerations, such as the scalability, electrochemical activity, etc., must be addressed when utilizing MXene as an active material. Thus, different modification strategies of MXene, such as surface termination, interlayer spacing, electrodes topologies, and constructing composites, are critical in determining the supercapacitor electrochemical performance. MXenes are mostly made by selective etching of their precursor layers, and the MA bond energy in various MAX-phases limits the etching environments. The bond energies of the 413 MAX (V_4_AlC_3_) phases, for example, are frequently higher than those of the 312 or 211 MAX (V_2_AlC) phases [168]. In order to etch a layer of the 413 MAX phase, this disparity leads to a longer duration and higher HF concentrations. The M_4_X_3_T*_x_* also has a low specific capacitance because of the moderately high molecular mass, and for that reason, it is rarely used as an electrode material. However, despite the multiple layers, V_4_C_3_T*_x_* exhibits poor performances. It has a higher capacitance than the delaminated V_2_CT*_x_* and the multi-layered Ti_3_C_2_T*_x_* and Ti_2_CT*_x_*. It is worth noting that, by increasing the “n” value of M_n+1_X_n_T*_x_* MXene, its cyclic stability can be remarkably improved. In fact, the Ti_3_C_2_T*_x_* MXene has the best electrochemical performance among all different types, although its fabrication method is too simple. Thus, it is of the utmost importance to investigate other types of MXenes. 

Ti_3_C_2_T*_x_* is the most-investigated MXene in supercapacitor applications [169]. The volumetric capacitance of the Ti_3_C_2_T*_x_* electrode in neutral, as well as basic, media was found to be 300–400F/cm^3^. Hence, the outstanding results outperform the current pure-carbon-based state-of-the-art EDLC and are even comparable to activated graphene-based electrode materials (i.e., capacitance 350F/cm^3^) [170]. Nevertheless, the cyclic voltammograms’ profile varies depending on the positive ions, referred to as the “capacitors” [171]. Subsequently, the protons, as the smallest cations, have contact with the largest number of electro-chemically active sites, and a volumetric capacitance (>900 F/cm^3^) was obtained in 1 M H_2_SO_4_ for pure laminated Ti_3_C_2_T*_x_* clay electrodes. The Li^+^ ions avoid the MXene sheets, assembling with interpolated H_2_O molecules during fabrication [172]. The MXene-based composites for supercapacitor applications are summarized in Table 6.

### 4.6. Cancer Therapy

**Figure 13:**
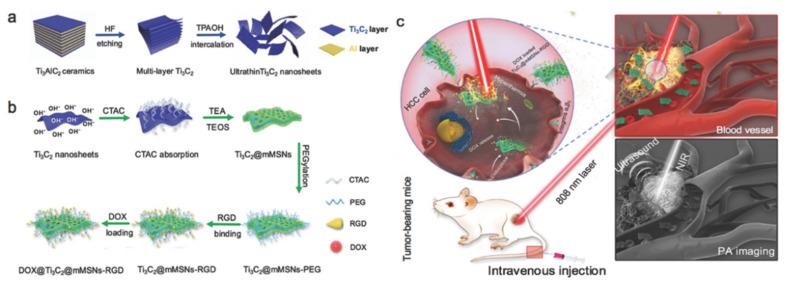
(**a**) Schematic of the fabrication of Ti_3_C_2_@mMSNs-RGD and the synergetic chemo-PTT against HCC with possible PA-imaging guidance and monitoring. (**a**) Schematic of the fabrication of Ti_3_C_2_ sheets. (**b**) Schematic of the fabrication of Ti_3_C_2_@mMSNs-RGD. (**c**) Schematic of the theranostic functions of Ti_3_C_2_@mMSNs-RGD. Reproduced with permission from ref. [187] Copyright 2018, Willey.

MXenes have recently been used as multifunctional biological material for a variety of purposes, including fluorescence imaging [57], computed-tomography imaging, photo-acoustic imaging [181], photo-thermal therapy, as well as photo-dynamic therapy [182,183]. The X-ray, PL, and near-infrared (NIR) light adsorption capabilities of MXenes are based on their tunable quantum confinement, localized-surface-plasmon resonance, as well as edge. The Ti_3_C_2_ MXene was firstly employed in photothermal therapy, which revealed an exceptional in vivo photothermal therapy efficacy. Since MXenes are composed of flexible elements that occupy the in-layer space, early translation metals could be chosen precisely to improve the element-based multi-functionalities of MXenes nanomaterials. As a rarely investigated MXene type, the two-dimensional Ta_4_C_3_ contains a biocompatible Ta element and has exceptional performance in the photothermal therapy of tumors. The Ta_4_C_3_ MXene has a broad absorption spectrum and exhibits a strong absorption band, almost similar to those of the two-dimensional graphene and MoS_2_ semiconductors. Thus, this material provides the necessary photo-absorption characteristics for the photothermal transduction process and exhibits the exceptional photothermal conversion efficiency of 44.7%, which is higher than most of the reported inorganic photothermal materials [184,185,186]. Some of the MXene-based nanomaterials were utilized as active theranostic platforms by integrating photo-acoustic computed tomography imaging with photo-thermal therapy. Integrating chemical therapy with photothermal therapy will help increase their cancer therapeutic efficiency even more. However, because there is no restricted region on the surface of MXenes, loading the medicine is problematic. Moreover, there is a lack of amine functionalities on MXenes for further functionalization, making the probable targeting alterations and surface engineering for precise goals such as target delivery/therapy more difficult. MXene coatings with biocompatible porous nanomaterials are one of the possible approaches in this scenario. For the first time, Li et al. [187] and his colleagues fabricated core@shell MXene/mesoporous silica. The silica layers with a mesoporous structure not only carry the therapeutic molecules DOX but also allow for the alteration of cancer cells using polyethylene glycol (PEG) and active target materials such as the three amino acid peptides, i.e., arginine, glycine, ad aspartic acid (RGD). They created a new theranostic nanoplatform, Ti_3_C_2_@mMSNs-RGD, by combining the photothermal effect of MXene (Figure 13a). DOX-Ti_3_C_2_@mMSNs-RGD exhibits pH- and NIR-activated release behavior (Figure 13b,c), and the acidity responsive characteristic allows for the controlled release of the DOX in tumors, where the micro-environment is slightly acidic (i.e., pH 5.8–7.1) and the intra-cellular environment is highly acidic (i.e., pH 5.0). Concurrently, the NIR-activated drug release is mostly restricted to the tumor cells, enhancing the outcome of chemical therapy. DOXTi_3_C_2_@mMSNs-RGD has a stronger cancer therapeutic impact than DOX, Ti_3_C_2_@mMSNs, and Ti_3_C_2_@mMSNs-RGD due to its targeted delivery capabilities. This work paves the road for aMXene/ordered mesoporous structure composite production and application. Afterward, these researchers improved the platforms of MXene and mesoporous silica by altering loading molecules and MXenes, thereby replacing the DOX with mesopores, creating agent such as CTAC to streamline the synthesis process and swapping Ti_3_C_2_ for Nb_2_C to obtain an NIR-II photothermal response [188]. They loaded the thermally decomposable radicals initiator into the mesopores to design a new thermodynamically cancer therapeutic system [189]. In fact, the MXenes’ combination with mesoporous materials offer a promising strategy for the design and fabrication of new emerging theranostic nanoplatforms for the efficient treatment of tumor cells.

## 5. Summary and Outlook

MXenes, or 2D transition metal carbides and nitrides, have received tremendous research attention in in the field of environmental science, biomedical technology, catalysis, energy conversion and storage, etc. The exceptional surface chemistry (i.e., termination with oxides and hydroxides functional groups), interesting optoelectronic properties, layered structure, and high charge carriers’ density of MXenes allowed for excellent performances in supercapacitors and next-generation energy conversion and storage devices. In summary, the unique features of MXenes and their prospective uses are mostly determined by manufacturing processes, structure, morphology, composition, and the number and variety of surface functionalities. Optimizing the synthesis techniques results in the improvement of MXenes with tunable and controllable properties. Progress, on the other hand, is foreseen, justifiable, and required to sustain the extension of MXenes. In the last five years, 2D carbides, nitrides, and carbonitrides have been fabricated by the selective exfoliation and etching of the layered ternary precursors, which resulted in a broad family of 2D MXenes. The hydrofluoric acid (HF) etching, modified-acid etching, molten salts etching, modified fluoride-based acid etching, and fluoride-free etching methods lead to the synthesis of MXenes with hydrophilic surfaces. In water-based colloidal solutions with high surface charges (i.e., negative zeta-potential greater than 30 mV), they are stable and do not need any surfactant for stabilization. This allows MXenes to be used in applications of device printing and coatings and films production. Furthermore, because of their exceptional physicochemical and opto-electronic features, MQDs have become a more popular branch of QD materials. Many investigations have revealed that QDs can be synthesized in a variety of methods. The top-down and bottom-up approaches are the most common in the synthesis of MQDs. The precursors of 2D MXenes were frequently industrialized by etching the MAX phases in high concentrations of acidic solutions (i.e., HF or LiF + HCl), which is highly toxic and harmful to human health. Thus, it is vital to explore the fluoride-free and environmentally benign etchants alternative to the conventional HF for MXenes fabrication. Meanwhile, there has been little investigation into bottom-up approaches for the fabrication of MQDs. Nevertheless, several of its benefits, such as the improved atomic usage, morphology and dimension modification, and better structural control, imply that it is a viable method to which more attention should be devoted. Furthermore, the stability and monodisperse nature are critical for improving the physicochemical features and practical use of MQDs, requiring the development of new technologies. Furthermore, precisely controlling the surface chemical properties of MQDs, as well as the formation of pure MQDs, is difficult. This is because that the fabrication process is mostly direct and suitable for adapting MQDs properties for specific applications. 

In addition, MXenes have been suggested to exhibit optical, thermoelectric, and mechanical capabilities in addition to their appealing structure. Even due to the existence of surface functionalities and H_2_O water molecules between their layers, Ti_3_C_2_T*_x_* has been found to have high metallic conductivity. On the other hand, Mo_2_CT*_x_* and Mo_2_TiC_2_T*_x_* behave similarly to semiconductors. For oxygen-terminated Ti_2_C, a bandgap of roughly 0.9 eV was estimated, and the Dirac cones are predictable for the fluorine-terminated Ti_2_C MXene. However, the prediction of many other properties is still awaiting experimental confirmation. 

Furthermore, the photocatalytic hydrogen generation over a Ti_3_C_2_ MXene photocatalyst provides new options for sustainable, clean, and renewable energy sources. Enormous efforts have been devoted to enhancing their solar-to-energy conversion efficiency. The unique structure and outstanding electrical features of Ti_3_C_2_ MXene allow it the distinctive ability for the generation of a Schottky barrier for electron trapping and employment as a cocatalyst to derive H_2_ generation. It is worth noting that the electrical and electronic features of Ti_3_C_2_ MXene play a vital role in boosting its efficiency. It is hypothesized that the incorporation of Ti_3_C_2_ MXene in various Ti_3_C_2_ MXene-based composites with metal–organic frameworks could remarkably enhance the physicochemical properties of the semiconductors in different ways, serving as effective support materials, lowering potential barriers, promoting carrier dynamics, and as electron trapping agents [190,191]. Thus, MXenes and MXene-based composite materials will have potential applications in various research fields including energy conversion and storage, catalysis, gas sensing, and phototherapy.

## 6. Future Perspectives

Future studies may comprise the effect of various precursor materials on the performance and fabrication of flawless surfaces of free functionality MXenes, the design and characterization of various carbide and nitride MXenes, and methods to optimize the top-down and the bottom-up approaches by selectively etching the layered precursors without the element’s “A”. 

In addition, understanding the impact of MXene’s structural design, such as the surface chemistry, interlayer gaps, and nanolayers stacking, on the charge storage mechanisms is of great significance. The process of increasing the gap between the nanolayers of MXenes should be further studied to promote the dynamics of ion diffusion and interpolation.

Further, the energy-storage mechanism of MXene-based materials is the main focus of future research, and the impact of thermal and chemical stability for energy storage performance is also of the utmost importance. The advancement in the fabrication of MXenes-based composites will reveal potential applications in the energy-storage field. Experimental studies on Ti_3_C_2_ MXene’s termination groups should be further extended. This is to clearly illustrate the importance of each terminal group in promoting MXenes’ stability and photocatalytic performance. For example, it should be clarified which terminating group is best suited to promoting the photocatalytic H_2_ evolution or other reactions.

For supercapacitor applications, the MXenes surface area is lower than that of graphene. If its specific surface area is brought to the level of graphene, this would likely increase its energy density and specific capacitance. The improvement can be made by adopting new approaches such as treatment with a concentrated acid and base to introduce pores. This would allow more space for hosting the pseudocapacitive particles and accommodate high-voltage electrolytic ions to enhance the energy density. The current surge in various metal (i.e., Li, Na, K, Zn) ions-based capacitors should be pushed forward to realize this high voltage.

It is investigated that the fabrication of MXenes is scalable, which means no property loss occurs when its batch size is increased from 1 to 50 g. However, it is of great significance to improve its batch size on the kilogram scale. For this, a chemical engineering technique should be designed to further scale-up MXenes’ production. Improvements should be made to yield precursors of the MAX phase in a cost-effective fashion. Additionally, cost-efficient synthesis methods should be utilized, i.e., self-propagating high-temperature fabrication from minerals or oxides precursors. 

For Ti-based MXenes (Ti, Al, C), the components are quite reasonable. However, for other MXenes (Ta, Hf, V), it is necessary to adjust the precursor cost, otherwise these MXenes will have limited industrial applications. It is vital to stress that MXenes are materials, not chemicals, and there is a direct relationship between their production and physicochemical properties, such as environmental stability and electrical conductivity. Thus, it is necessary to understand the relationship between the fabrication and processing of the MAX-phase precursors and MXenes properties. The current state-of-the-art can be upgraded by recognizing less aggressive etchants and accelerating the fabrication process of MXenes.

Through operand characterization techniques, Ti_3_C_2_ MXene matrix materials can be deeply investigated, thereby obtaining basic knowledge of the relationship between the molecular structure and activity of Ti_3_C_2_ MXene and its selectivity. This technology can conduct extensive research on the stability of Ti_3_C_2_ MXenes under different reaction conditions, understand the properties of the catalytic site of Ti_3_C_2_ MXenes’ reaction mechanism, and help improve the role of Ti_3_C_2_ MXene in the field of photocatalysis.

## Figures and Tables

**Table 1:** Porous MXenes, synthesis methods, and their structures [15].

Samples	Methods	Structures
Porous Ti_3_C_2_T*_x_* Aerogel shaped	Freeze drying	Mesoporous/macroporous
MXene lamellar-liquid-crystal	Mechanically shearing assisted freeze drying	Aligned vertically mesoporous/macroporous
Lamellar structured Ti_3_C_2_T*_x_*/SiCnws foam	Bidirectional freeze-drying	Aligned Parallelly mesoporous/macroporous
Super-elastic MXene/PI aerogels	Freeze drying	Meso/macropore with wide size distribution
Fluffy-type MXene microspheres	Spray drying	Mesoporous/macroporous
3D MXene films	Hard-template method	Mesoporous/macroporous
Cellular-type MXene foam	Hydrazine reduction technique	Mesoporous/macroporous
hybrid 3D porous network of MXene-Sponge	Dip-coating and drying	Macroporous
MXene-rGO aerogel	Chemical-reduction	Mesoporous/macroporous
MXene-rGO aerogel	Freeze drying-calcination	Mesoporous/macroporous
Cellulose-MXene aerogel	Chemical crosslinking	Mesoporous/macroporous
TiO_2_/MXene, SnO_2_/MXene	Self-assembly	Mesoporous
FeNi-LDH-MXene	In-situ growth	Mesoporous
Core-shell Ti_3_C_2_-mSiO_2_	Sol-gel	Mesoporous
MXene flakes	Oxidative-etching	Mesoporous
MXene with divacancy-ordering	Selective-etching	Microporous and mesoporous

**Table 2:** MXenes with various compositions have varying mechanical characteristics [93].

Materials	Morphology	Elastic Constants c11 [GPa]	Young’s Modulus E [GPa]	Strains aAong Uniaxial x (εx)	Strengths Along Uniaxial x (σx) [GPa]
Ti_3_C_2_H_2_	2D unit cell	419	392	-	-
Zr_3_C_2_O_2_	2D-hexagonal lattice	392.9	-	-	-
Ti_2_C	2D sheets	609	-	-	-
Ti_2_CO_2_	2D sheets	-	983	-	-
Ta_2_C	2D sheets	788	-	-	-
Ti_2_C	2D sheets	-	597	-	-
Mo_2_C	2D sheets	-	312	-	-
Ti_2_CO_2_	2D sheets	745	570	0.28	56
W_2_C	2D sheets	781.9	-	0.16	65.6
Materials	Morphology	c11 (N/m)	E (N/m)	Εx	σx (N/m)
Ti_3_C_2_O_2_	2D unit cell	379	347	-	-
W_2_HfC_2_O_2_	2D unit cell	-	-	-	47.3
Mo_2_CO_2_	hexagonal unit cell	361	302	-	-
Ti_2_CO_2_	2D sheets	-	241	0.24	30.7

**Table 3:** Summary of the photocatalytic performance of MXene-based photocatalysts.

Photocatalysts	Activities	Light	Ref.
Ti_3_C_2_/g-C_3_N_4_	H_2_ evolution	Visible light	[126]
ZnIn_2_S_4_/Ti_3_C_2_T_X_	H_2_ evolution	Visible light	[127]
MXene/Zn_x_Cd_1−*x*_S	H_2_ evolution		[128]
Ti_3_C_2_/g-C_3_N_4_	CO_2_ conversion	Visible light	[129]
Ti_3_C_2_/Bi_2_WO_6_	CO_2_ conversion	Simulated light	[130]
Ti_3_C_2_/B-doped g-C_3_N_4_	CO_2_ conversion	Visible light	[131]
TiO_2_/Ti_3_C_2_T*_x_*	Benzene removal	Near IR	[132]
Ti_3_C_2_/g-C_3_N_4_	Methylene blue degradation	Visible light	[133]
Ti_3_C_2_/MoS_2_	Methyl orange degradation	Visible light	[134]
Ti_3_C_2_/SnNb_2_O_6_	Tetracycline hydrochloride removal	Visible light	[135]

**Table 4:** Summary of the HER performances of MXene-based electrocatalysts [139].

Electrocatalysts	Electrolytes	Overpotential at 10 mA·cm^−2^	Tafel slope (mV·dec^−1^)
Ti_3_C_2_O*_x_*	0.5 M H_2_SO_4_	190	60.7
Ti_3_C_2_(OH)*_x_*	0.5 M H_2_SO_4_	270	88.5
Ti_3_C_2_T*_x_*-450	0.5 M H_2_SO_4_	266	109.8
Mo_2_TiC_2_T*_x_*–Pt_SA_	0.5 M H_2_SO_4_	30	30
Mo_2_CT*_x_*: Co	1 N H_2_SO_4_	180	/
Mo_2_CT*_x_*	1 N H_2_SO_4_	230	/
Ti_3_C_2_T*_x_* nanofibers	0.5 M H_2_SO_4_	169	97
Ti_3_C_2_T*_x_* flakes	0.5 M H_2_SO_4_	385	188
Pt/Ti_3_C_2_T*_x_*	0.1 M HClO_4_	32.7	32.3
NiCo@Nb–Ti_3_C_2_T*_x_*	1 M KOH	43.4	116
MoS_2_/Ti_3_C_2_T*_x_*@C	0.5 M H_2_SO_4_	135	46
NiSe_2_/Ti_3_C_2_T*_x_*	0.5 M H_2_SO_4_	200	23.7
NiS_2_/V_2_CT*_x_*	1 M KOH	179	85
MoSe_2_/Ti_3_C_2_T*_x_*	1 M KOH	95	91
MoS_2_/Ti_3_C_2_T*_x_* nanoroll	0.5 M H_2_SO_4_	152	70
VS_2_/V_2_CT*_x_*	1 M KOH	164	47.6
BP QDs/Ti_3_C_2_T*_x_*	1 M KOH	190	83
FeNi@Mo_2_TiC_2_T*_x_*@NF	0.5 M H_2_SO_4_	165	103

**Table 5:** Summary of the MXene-based composites for gas sensing applications.

MXenes Composities	Types of Gases	Temperature	Concentration	Response (%)	Ref.
polypyrrole/Ti_3_C_2_T*_x_*	NH_3_	Room temperature (RT)	100 ppm	31.9	[157]
MXene-Polymer	NH_3_	RT	500 ppb	2.2	[158]
In_2_O_3_/Ti_3_C_2_T*_x_*	Methanol	RT	5 ppm	29.6	[159]
Ti_3_C_2_T_x_/WSe_2_	Ethanol		40 ppm	0.24	[160]
Ti_3_C_2_/TiO_2_	NO_2_	RT	5 ppm	1.13	[161]
Ti_3_C_2_–MoS_2_	NO_2_	RT	100 ppm	72.5	[162]
Ti_3_C_2_ MXene-based	NH_3_	RT	500 ppm	6.13	[163]

**Table 6:** Summary of the MXene-based composites for supercapacitor applications.

MXenes Based Composites	Specific Capacitance	Electrolyte	Performance	Stability	Ref.
Ti_3_C_2_T*_x_*/polyaniline	556.2 F g^−1^ at 0.5 A g^−1^	H_2_SO_4_ (1 M)	437.7 F g^−1^ at 5 A g^−1^	91.6% at 5 A g^−1^ (5000 cycles)	[173]
WO_3_–Ti_3_C_2_	566 F g^−1^	H_2_SO_4_ (1 M)		92% (5000 cycles)	[174]
Ti_3_C_2_–Cu	885.0 F g^−1^ at 0.5 A g^−1^	H_2_SO_4_ (1 M)	131.6 F g^−1^ at 10 A g^−1^ (1 M KOH)	89% at 2 A g^−1^ (10,000 cycles)	[175]
Ti_3_C_2_/TiO_2_	102.5 F·g^−1^ at 300 mA·g^−1^	H_2_SO_4_ (1 M)		89.1% (2500 cycles)	[176]
PANI/V_2_C	337.5 F g^−1^ at 1 A g^−1^	H_2_SO_4_ (1 M)	292.5 F g^−1^ at 20 A g^−1^	97.6% at 5 A g^−1^ (10,000 cycles)	[177]
Ti_3_C_2_/CNTs/MnCo_2_S_4_	823 F g^−1^ at 1A g^−1^	H_2_SO_4_ (1 M)		94.09% (5000 cycles)	[178]
MXene/graphene	393 F g^−1^ at 2 mV s^−1^	H_2_SO_4_ (3 M)	129 F g^−1^ at 10 V ^s−1^	95.7% at 200 mV s^−1^ (10,000 cycles)	[179]
Ti_3_C_2_–MoS_2_	1459 F g^–1^ at 1 A g^–1^	H_2_SO_4_ (1 M)		90% (3000 cycles)	[180]

## Data Availability

Not applicable.
